# Digital hydraulic valves: Advancements in research

**DOI:** 10.1016/j.heliyon.2024.e27264

**Published:** 2024-03-03

**Authors:** Francesco Sciatti, Paolo Tamburrano, Elia Distaso, Riccardo Amirante

**Affiliations:** Department of Mechanics, Mathematics and Management (DMMM), Polytechnic University of Bari, Bari, Italy

## Abstract

Despite the great advantages it offers, such as high-power density, precise control and large force output, conventional hydraulic valve control suffers from low energy efficiency due to substantial energy losses that occurs as the pressurized oil flows through the hydraulic circuit and its components, particularly the control-ones. Conventional hydraulic technology typically utilizes analogue spool valves, such as proportional and servovalves, as control components in various industrial and aeronautical applications where high precision and fast response are required. However, the use of these valves leads to high power dissipation, caused by the significant pressure drop across the small narrow passages uncovered during valve control. To maintain the relevance of hydraulic technology across industries, researchers are investigating a novel research field, also referred to as digital hydraulic technology. By using some digital concepts into hydraulic technology, the goal of this innovative research field is to replace conventional analogue spool valves with robust and low-cost digital On/Off valves, thus minimizing power losses and enhancing the overall efficiency of hydraulic systems. This paper presents a thorough review of the research advancements in digital hydraulic technology, with a specific focus on valve control. Firstly, the standard definition of digital hydraulic technology and its two main categories are introduced. Then, the operating principles and the digital approaches used to control digital hydraulic valves are examined. Afterwards, the digital hydraulic valve architectures developed over the years are reviewed, along with an in-depth analysis of their performance. Finally, the potential application scenarios, advantages and challenges with digital hydraulic valves are explored, highlighting potential areas for future research and development.

## 1 Introduction Conventional hydraulic technology

**Fig. 1:**
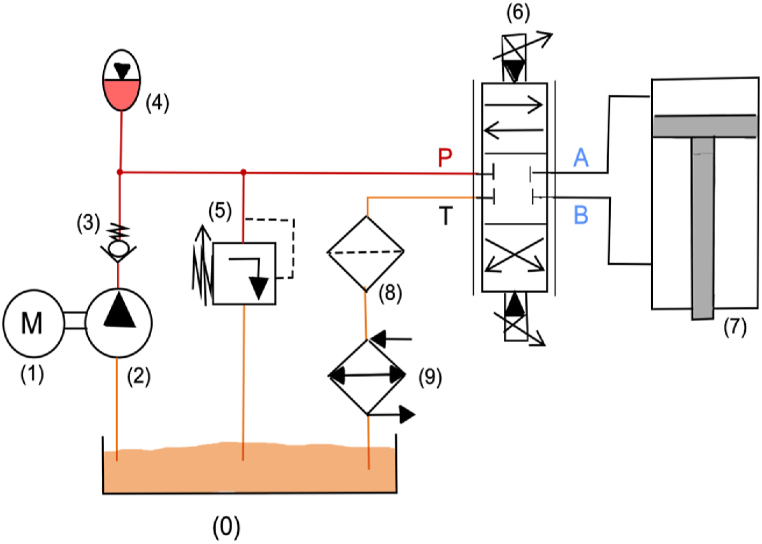
Conventional hydraulic circuit: (**1**) Prime Mover; (**2**) Hydrostatic Pump; (**3**) Check Valve; (**4**) Accumulator; (**5**) Pressure Relief Valve; (**6**) Electrohydraulic Servovalve; (**7**) Linear Actuator; (**8**) Filter; (**9**) Heat Exchanger.

Fluid power is a technology based on the use of pressurized fluids, including liquids (hydraulics) or gases (pneumatics) to generate, control, and transmit power. The basics of modern fluid power systems date back approximately 350 years ago to the discoveries of Blaise Pascal (Pascal's Law) and Daniel Bernoulli (Bernoulli's Principle) [1]. These studies enabled Joseph , an English inventor and locksmith who is considered the grandfather of modern hydraulic technology, to patent the first hydraulically operated machine, “The Hydraulic Press,” in 1795 [2]. With the advent of the Industrial Revolution, hydraulic technology experienced a strong growth thanks to the development of several hydraulic components, which led to modern hydraulic technology [3]. Fig. 1 shows the hydraulic schematic of a conventional hydraulic system with valve control, where the hydraulic components needed to realize an exemplary hydraulic circuit are represented using ISO (International Organization for Standardization) symbols.

Over the past few decades, engineers and researchers worldwide have faced the main challenge of realizing progressively efficient hydraulic components. Of all the hydraulic components, the control-ones are the most critical since they have a great impact on the overall energy efficiency of hydraulic systems. Control valves play an important role in hydraulic systems as they manage hydraulic power by regulating the mechanical power transmitted to an actuator. This control can be obtained by managing pressure (using pressure control valves), controlling flow rate (with flow control valves), or directing flow (via directional control valves) [4]. The latter are available with different performance and characteristics depending on the application. In most cases, the higher the performance, the higher the cost of the valves.

Conventional hydraulic systems typically use analogue spool valves, such as proportional and servovalves, as directional control valves in a variety of industrial and aeronautical applications that require high precision and fast response [[5], [6], [7], [8], [9]]. This holds true for pneumatic systems as well. For instance, in vital biomedical equipment like lung ventilators for intensive care units, analogue spool valves are utilized to achieve precise control of the gas being inhaled or exhaled by the patient [10]. These valves, indeed, unlike On/Off valves, allow for precise control of an actuator position and/or velocity without causing rapid acceleration or deceleration [11]. Therefore, the flow of pressurized fluid delivered to an actuator, and consequently its position and velocity, can be easily controlled based on the input electrical signal to the valve [11]. A comprehensive review of the state of the art and the research progress of both directly driven proportional hydraulic valves and electrohydraulic servovalves is provided in Refs. [12,13], respectively.

Electrohydraulic servovalves are mainly classified in two main types. The single-stage type, in which the sliding spool is directly controlled by integrated electronics using a linear force motor, and the double-stage type, in which the sliding spool is housed in the main stage and is controlled indirectly by the electronics through the pilot stage [13,14]. The latter type can be equipped with a centring spring for mechanical feedback or with a Linear Variable Differential Transformer (LVDT) sensor for electrical feedback [13,14]. In contrast, proportional valves are generally a single-stage configuration, in which the sliding spool is centered by means of two springs within the valve body and is directly moved by two proportional solenoids positioned on opposite sides of the valve [12]. In-depth analyses are available to examine the fluid dynamic behaviour of commercial hydraulic proportional valves [15,16]. These analyses provide the means to evaluate the effect of the flow forces and of the cavitation phenomenon on the performance of this kind of spool valves.

**Fig. 2:**
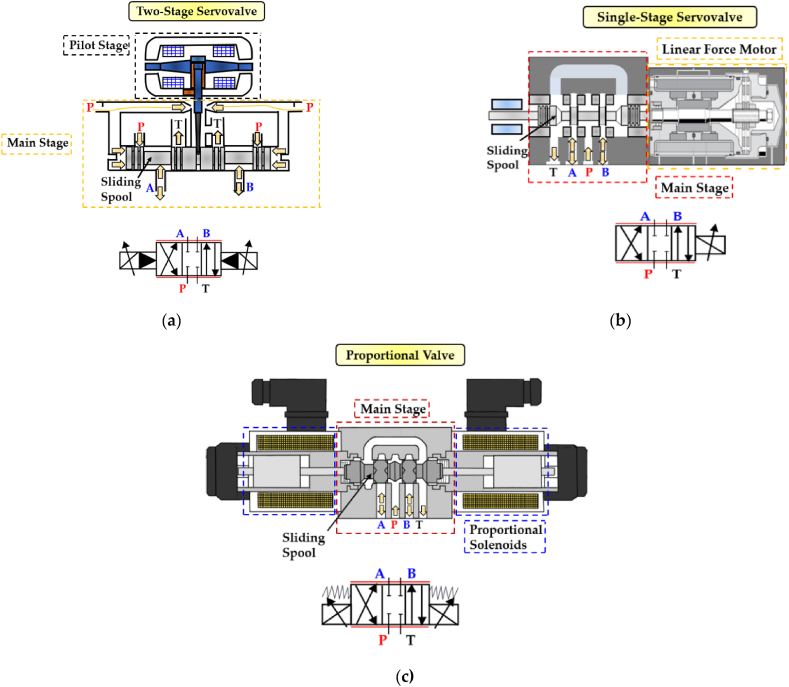
Conventional analogue spool valves and their relative ISO symbol: (**a**) Double-Stage Servovalve; (**b**) Single-Stage Servovalve; (**c**) Proportional Valve.

A key difference between proportional and servovalves (both single-stage and double-stage) is the use of a bushing sleeve in the latter. This component enables servovalves to achieve finer tolerances by reducing the overlap between spool lands and slots, resulting in more precise control [12,13]. A picture depicting the conventional analogue spool valves is shown in Fig. 2.

The summarized performance of these four-way three-position (4/3) conventional analogue spool valves can be found in Table 1 [17]. Among them, double-stage servovalves stand out for their exceptional performance characteristics, which include high-actuation forces [13], excellent accuracy [18], precise controllability [19], rapid response times [20], high-bandwidth and low weight [13]. However, it is important to note that a faster valve is not always a benefit, it depends on the hydraulic circuit and its use cases.

**Table 1:** Typical performance of conventional analogue (4/3) spool valves, operating at 40 L/min with 70 bar pressure drop [17].

	Proportional Valve	Single-Stage Servovalve	Double-Stage Servovalve (Mechanical Feedback)	Double-Stage Servovalve (Electrical Feedback)
Actuation Force	∼50 [N]	∼200 [N]	∼500 [N]	∼500 [N]
Step Response (100%)	50 [ms]	15 [ms]	10 [ms]	3 [ms]
90-degree phase lag frequency	10 [Hz]	50 [Hz]	100 [Hz]	200 [Hz]
Cost	low	medium	high	very high
Size	very large	very large	small	medium

Despite the advantages they provide, double stage servovalves are criticized for their susceptibility to impurities and relatively high cost [13,21]. Moreover, conventional analogue spool valves, both proportional and servovalves, are associated with significant energy losses, primarily caused by two factors:

1. The internal leakage ($Q_{1,L}$) that occurs in the main stage due to necessary geometrical tolerances for spool movement, such as the radial clearance between the sliding spool and the valve body or the bushing sleeve (in the case of proportional or servovalves, respectively) [22,23];
2. The high pressure drops (*Δp*) that occur when pressurized oil flows through the small passages uncovered by the sliding spool during valve control [13].

Additionally, another cause of power consumption occurs for double-stage servovalves:

3. The continuous and constant quiescent oil flow ($Q_{2,L}$) required in the pilot stage even when the sliding spool is in the neutral position [13].

**Fig. 3:**
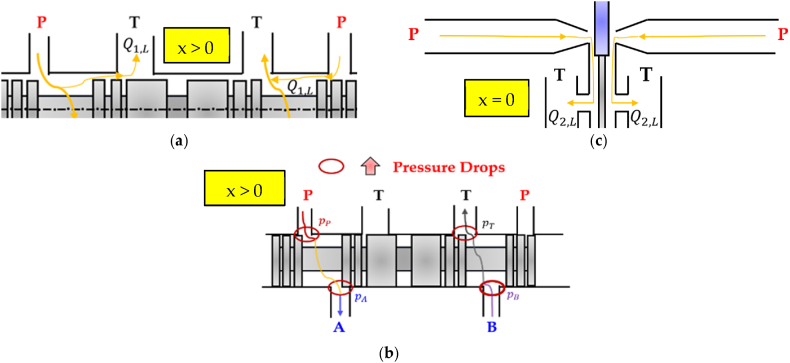
Main power losses in a conventional double-stage servovalve: (**a**) Internal Leakage of the main stage; (**b**) High pressure drops due to the small passages of the main stage; (**c**) Internal Leakage of the pilot stage.

In order to provide a better explanation of the energy losses that occur within conventional spool valves, Fig. 3 depicts a sliding spool in motion (x > 0) and in the neutral position (x = 0) within the bushing sleeve of a generic four-way three-position (4/3) servovalve, where “x" represents the sliding spool displacement.

Double-stage servovalves commonly exhibit significant internal leakage in the pilot stage. For instance, in the case of a Moog double nozzle-flapper servovalve (series 30) at an inlet pressure of 210 bar, the quiescent flow is approximately 0.73 L/min, leading to substantial power consumption [24]. Additionally, the internal leakage occurring in the main stage of a four-way three position (4/3) spool valve, with zero overlap between the sliding spool and the bushing sleeve, was investigated in Ref. [25]. The investigation revealed that the amount of leakage flow varies throughout the entire stroke range of the sliding spool, depending on the supply pressure and oil viscosity. Specifically, at a supply pressure of 250 bar and with an oil temperature of 45 °C, the leakage flow measured when the sliding spool was in the neutral position was approximately 0.8 L/min.

The power consumption resulting from the internal leakage of both the main stage and the pilot stage has been the topic of several research studies. Regarding the former, researchers have found that geometrical defects, due to the wear and manufacturing processes, have a considerable impact on the internal leakage of the main stage [26,27]. Concerning the latter, various prototypes of novel pilot stages have been developed using piezoelectric actuators [[28], [29], [30]]. It has been proven that the use of novel piezo-valves, i.e., valves actuated by piezo-electric actuators, can drastically minimize the energy consumption caused by the internal leakage of the pilot stage, while also improving response time and reducing both the complexity and cost of the valve [28,29]. A detailed review of innovative architectures of electro-hydraulic servovalves that exploit actuation systems based on piezo-electric materials can be found in Ref. [24].

Despite the significant improvements achieved in recent years, conventional analogue spool valves continue to experience high energy dissipation due to the second source of energy losses mentioned above. Indeed, the spool architecture of these valves leads to significant energy losses. This occurs because when the pressurized fluid passes through the narrow passages formed by the sliding spool during valve control, it encounters significant pressure drops [12,13]. In Ref. [31], a medium-sized four-way three-position (4/3) analogue spool valve was examined to assess energy losses. When both metering chambers were simultaneously opened (P → A and B → T for sliding spool right displacement or P → B and A→T for sliding spool left displacement), with a 30 bar pressure drop, a flow rate of 60 L/min and considering a pump efficiency of 80%, it was found that the valve dissipated approximately 7 kW of power.

To maintain the relevance of hydraulic technology across industries, in recent years there has been a significant increase in interest in the emerging field of digital hydraulic technology. This novel technology aims to replace conventional analogue spool valves, both proportional and servovalves, with low-cost and robust digital On/Off valves known as digital hydraulic valves. By employing the On/Off design, these valves can adopt a structure similar to that of poppet valves, allowing for a larger flow area and a reduced pressure drop. Consequently, this technology has the capacity to minimize energy losses and improve the overall energy efficiency of hydraulic systems [[32], [33], [34]]. The promising results achieved from a range of applications, including aeronautical and industrial sectors [[35], [36], [37], [38]], have underscored the potential of digital hydraulic technology to revolutionize the field of fluid power, enabling the efficient realization of modern and energy-efficient hydraulic systems [39].

In this scenario, given the ever-increasing importance of digital hydraulics, the purpose of this paper is to provide a comprehensive review of the current state-of-the-art and research progress in digital hydraulic technology, with a particular focus on digital hydraulic valves. Since this is an emerging field and previous definitions have only partially captured its characteristics, the paper begins by introducing the standard definition of digital hydraulic technology and its two main branches, i.e. the high frequency switching and parallel digital hydraulic technologies (Section 2, Digital Hydraulic Technology). Subsequently, it explores the operating principles and digital approaches employed in controlling digital hydraulic valves, highlighting the strengths and weaknesses of each method (Section 3, Digital Hydraulic Valves). Following this, the paper examines the architectures of digital hydraulic valves developed, over the years, by researchers and companies offering an in-depth analysis of their performance (Section 4, Research Advancements in Digital Hydraulic Valves). Finally, the analysis concludes by delving into potential application scenarios, advantages over conventional analogue spool valves, and the associated challenges, thereby identifying areas for future research and development (Section 5, Challenges and Future Directions in Digital Hydraulic Technology).

## 2 Digital hydraulic technology

As computer and microelectronic technology are increasingly used in industrial and aeronautical applications, the trend towards digitalization has become inevitable in the progress of hydraulic technology. The outstanding results achieved in information, communication, and power electronics, coupled with the similarities between electrical and hydraulic systems, demonstrate that use of digital concepts into hydraulic technology could be instrumental to address the energy issues faced by conventional hydraulic systems [32,34]. The term “digital hydraulic technology” was first introduced more than a decade ago by Matti Linjama [33]. Over the last years, this novel technology has gradually gained recognition as a distinct branch of fluid power, particularly in research fields. Alongside the traditional institutes in Tampere (TUT/IHA) [40], and Linz (JKU/IMH/LCM) [41], several other European research institutions, such as Bath University (PTMC) [42], RWTH Aachen (IFAS) [43], and Aalborg University (AAU/ET) [44], have focused their attention on various research related to digital fluid power.

**Fig. 4:**
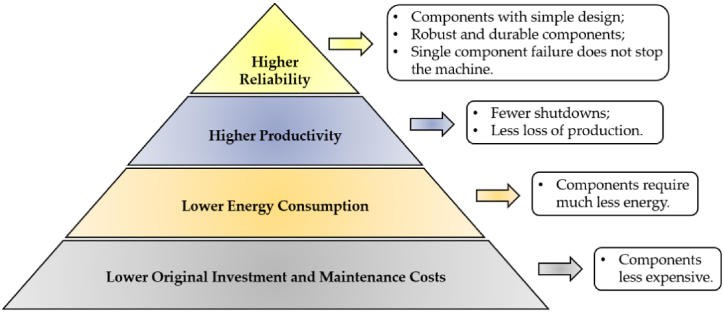
Advantages of digital hydraulic technology compared to conventional hydraulic technology, adapted from Ref. [48].

Although digital pneumatic systems have been reported in literature [[45], [46], [47]], the majority of digital fluid power systems that have been studied so far are hydraulic. This is mainly because digital hydraulic technology offers significant advantages compared to conventional hydraulic technology, including higher reliability, lower energy consumption, greater precision in machine movements, fewer shutdowns, less production loss, higher component standardization, and lower original investment and maintenance costs [[48], [49], [50], [51]]. Fig. 4 illustrates the advantages of digital hydraulic technology over conventional hydraulic technology.

The standard definition of digital hydraulic technology, namely “a system which controls a discrete fluid with a modulated, discrete, digital signal directly to realize active and intelligent control of the system output”, was provided only four years ago in Ref. [52]. The authors noted that the previous definitions by M. Linjama [53] and H. Yang [54] only partially captured the characteristics of digital hydraulic technology. Based on the new definition, hydraulic components with the ability to discretize fluid flow or control signals are referred to as digital hydraulic components, while hydraulic systems composed of such components are known as digital hydraulic systems. Interestingly, the idea that discrete control could offer advantages over continuous control was already discussed in the 19th century [[55], [56], [57]].

The large variety of the digital hydraulic systems can be classified into two main categories [53]:

1. High frequency switching digital hydraulic systems;
2. Parallel digital hydraulic systems;

High-frequency switching digital hydraulic technology employs components that switch rapidly and continuously over time, allowing active and intelligent control of the system's output [52]. These systems can be classified as temporal output discrete systems [58,59]. To control the opening and closing of the components and achieve high-frequency switching, pulse width modulation techniques (PWM) are employed, allowing the system to attain different discrete output values [52]. Theoretically, these systems can achieve any value within a specific range, although the output remains discrete due to the switching frequency of the components [52]. The latter significantly impacts the system's performance, as lower switching frequencies provide better control but lead to increased pressure pulsations [53]. This technology finds practical use in digital hydraulic buck converters (DHBCs) [60], antilock braking systems (ABSs) [61], and fuel injectors [62].

On the other hand, parallel digital hydraulic technology involves connecting digital hydraulic components in parallel to actively and intelligently regulate the system's output [52]. These systems can be classified as spatial output discrete systems [58,63]. By employing digital coded signals, such as binary scheme, they can manage the opening and closing states of the parallel-connected components, allowing for different state combinations and enabling the system to achieve various discrete output values [52]. Furthermore, since these systems can attain a specific number of discrete output values, determined by the number of parallel-connected components, the need for frequent “on/off” switching of the components becomes unnecessary [53]. In comparison to high-frequency switching digital hydraulic systems, parallel digital hydraulic systems, used in applications such as digital flow control units (DFCUs) [64], digital hydraulic power management systems (DHPMSs) [65], and digital hydraulic hybrid actuators (DHHAs) [66], offer enhanced scalability, programmability, and reliability [67].

## 3 Digital hydraulic valves

**Fig. 5:**
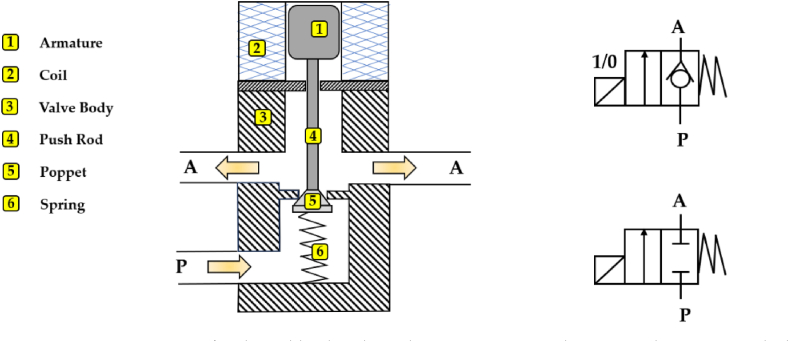
A two-way two-position (2/2) digital hydraulic valve (poppet-type design) and its ISO symbols.

Digital hydraulic valves are the core components in digital hydraulic technology, and their characteristics have a great impact on the performance of the entire digital hydraulic system. These valves, being On/Off, may not require a spool for flow adjustment and can have the same architecture as that of poppet or ball valves. The latter are capable of performing several working cycles with lower pressure drops than analogue and fragile spool valves because the flow area can be designed larger [31]. The main difference between a ball valve and a poppet valve is that the former may experience leakage due to the gap between the ball and the valve seat, while the latter ensures no leakage. A cross-section view and the relative ISO symbols of a two-way two-position (2/2) digital hydraulic valve with a poppet-type design are shown in Fig. 5.

As shown in Fig. 5, the digital hydraulic valve is composed of an armature (1), a coil (2), a valve body (3), a push rod (4), a poppet (5) and a spring (6). Regarding its operation, when the coil is not energized, the absence of electromagnetic force causes the poppet to remain in close contact with the valve seat, thanks to the combined action of the spring force and hydraulic force. Conversely, upon energizing the coil, the progressively rising of the electromagnetic force overcomes the cumulative spring force and hydraulic force, initiating the gradual opening of the valve.

The use of digital On/Off valves rather than conventional analogue spool valves, including proportional and servovalves, results in many advantages for digital hydraulic technology [33,53]:

• Simpler and less expensive valves;
• Simpler control electronics;
• Higher robustness;
• Lower energy consumption;
• Longer lifetime;
• Higher flexibility;
• Easy connection with computers and PLCs;
• Higher safety and reliability;
• Less sensitive for contamination;
• No need for spool position feedback;
• No leak if poppet type valves are used.

In terms of this last point, proportional valves are designed with appreciable overlap (greater than 5% of the sliding spool stroke), leading to minimal leakage [12,13]. In contrast, the sliding spool overlap in servovalves is usually very small (often 1% of the sliding spool stroke or less [68]), resulting in high leakage [12,13].

The following Table 2 presents the commercially available On/Off valves designed for digital hydraulic applications [69].

**Table 2:** Commercially available On/Off valves for digital hydraulic applications [69].

	SUN DLV	Rexroth SEC6	Rexroth WES	Parker GS02-73	LCM FSVi4.1	Bucher WS22GD
					
**Response Time [ms]**	10	7–10	5	5	<3	5–30
**Maximum Flow Rate [L/min] (@ Pressure drop [bar])**	1 (350)	25 (420)	200 (350)	- (210)	25 (300)	30 (350)
**Switching Frequency [Hz]**	13	40	10	–	200–500	–
**Price [€]**	∼100	∼600	–	∼70	∼1500	∼150

**Fig. 6:**
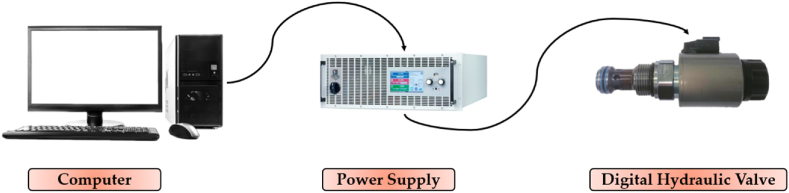
A scheme representing the control of digital hydraulic valves.

Digital hydraulic valves can be controlled using digital control methods, which involve generating a Digital Command Signal (DCS) from a computer or controller to activate or deactivate the valve. The DCS is then amplified through an electronic power supply and used to drive the digital hydraulic valve, as shown in Fig. 6. Digital approaches are commonly used for controlling digital hydraulic valves because the latter, being On/Off, have only two steady states that can be compared with the “one” and “zero” of digital control methods. According on the different control approach, digital hydraulic valves can be further classified into [52]:

1. High Frequency Switching Digital Hydraulic Valves;
2. Parallel Digital Hydraulic Valves.

**Fig. 7:**
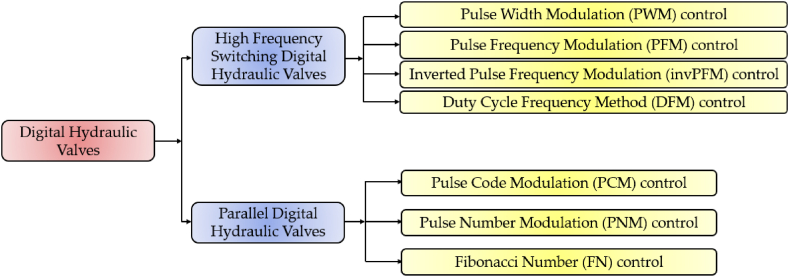
Classification of digital hydraulic valves based on the different control approaches involved.

The digital control approach employed depends on both the specific application requirements and the characteristics of the valve being used. To illustrate this, Fig. 7 presents a classification of digital hydraulic valves based on the different digital control approaches involved.

### 3.1 High frequency switching digital hydraulic valves

Switching technologies are a type of digital hydraulic systems that involve the use of high frequency switching On/Off valves (HFSVs). These valves are commonly known as pulse modulation switching digital hydraulic valves since they use pulse modulation techniques to control the flow of hydraulic fluid. Among the pulse digital control approaches, the Pulse Width Modulation (PWM), the Pulse Frequency Modulation (PFM), the inverted Pulse Frequency Modulation (invPFM) and the Duty Cycle Frequency Method (DFM) are the most common employed to adjust the valve's output [70]. To differentiate between the various pulse digital control methods, it is necessary to examine the DCS used for controlling the poppet position of the HFSV. In pulse control signals, the DCS is an alternating signal that switches between “logical one” and “logical zero” states, which are represented by the pulse duration $\left(t_{ON}\right)$ and the pause time $\left(t_{OFF}\right)$, respectively [70]. The sum of the pulse duration $\left(t_{ON}\right)$ and the pause time $\left(t_{OFF}\right)$ gives the overall period (τ) of the DCS:

(1) $$\tau =t_{ON}+t_{OFF}$$

To describe the amount of the pulse duration $\left(t_{ON}\right)$, the concept of duty cycle is introduced. The duty cycle, denoted by (*%DC)*, is the ratio of the pulse duration $\left(t_{ON}\right)$ to the overall period (τ) of the DCS, as evaluated in the following equation:

(2) $$\left(\%DC\right)=\frac{t_{ON}}{\tau }$$

Since the previous control approaches are limited to a single control variable, specifically the pulse duration ($t_{ON}$) or the pause time ($t_{OFF}$), a third equation is necessary to differentiate the DCS among the main pulse digital control approaches, as shown in Table 3 [[70], [71], [72]].

**Table 3:** Pulse digital control approaches used to control HFSVs [[70], [71], [72]].

Pulse Digital Control Approach	Control Variable	Third Equation	Variables Calculated
PWM	$t_{\text{ON}}$	$\tau =\frac{1}{f}=\text{const.}$	$t_{\text{OFF}}-\;\left(\%\text{DC}\right)$
PFM	$t_{\text{OFF}}$	$t_{\text{ON}}=\text{const}$.	f
invPFM	$t_{\text{ON}}$	$t_{\text{OFF}}=\text{const}$.	f
DFM	$t_{\text{ON}}$	(%DC)=const.	$t_{\text{OFF}}-$ f

The PWM method controls the pulse duration ($t_{ON}$), while keeping the period (τ) or the switching frequency (f = 1/τ) constant, in order to modify the duty cycle (%DC) of the DCS. In contrast, the other three digital control approaches, namely the PFM, the invPFM and the DFM, can be used to manage the frequency (f) of the DCS.

**Fig. 8:**
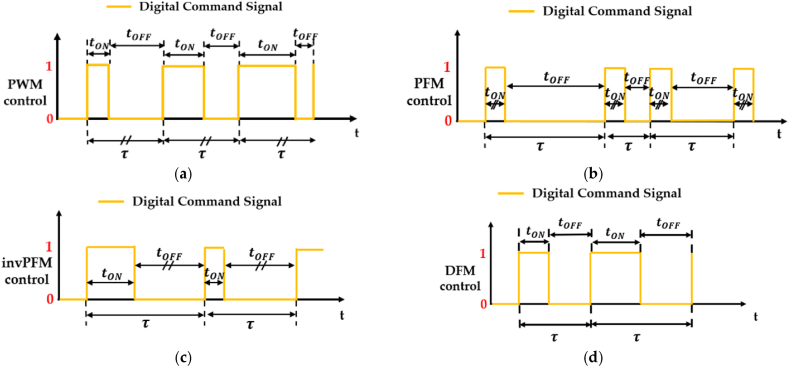
Differences between the digital command signal used to control HFSVs: (**a**) PWM; (**b**) PFM; (**c**) invPFM; (**d**) DFM, adapted from Refs. [[70], [71], [72]].

An illustration of the DCS used to control HFSVs with these four pulse digital control approaches is showed in Fig. 8.

**Fig. 9:**
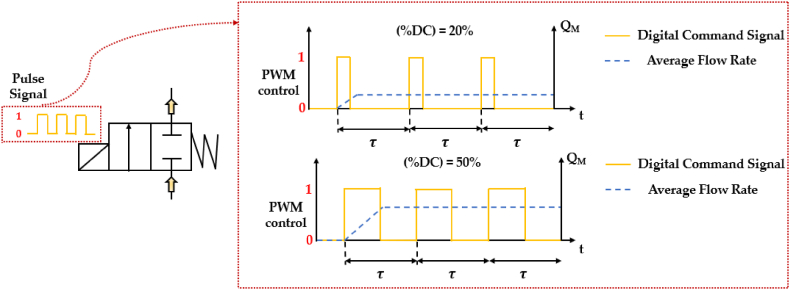
Two way two-position (2/2) HFSV controlled with the PWM control approach.

The use of different pulse modulation techniques allows for the description of the working principle of a two-way two-position (2/2) HFSV. As shown in Fig. 9, a pulse control signal (from 0 to 1) is delivered to the HFSV and flow modulation is obtained by adjusting the frequency (through PFM, invPFM or DFM controls) or the duty cycle (through PWM control) of the DCS. The controllability of the valve is influenced by the switching frequency. A lower switching frequency results in higher controllability, but it also leads to an increase in pressure pulsation and a decrease in the average flow rate obtained. To suppress noise and achieve a smoother flow rate, accumulators and inertance tubes are commonly employed [73]. In the graph, the average flow rate provided by the 2/2 HFSV is represented by the symbol (Q_M_) on the right y-axis.

The behaviour of an HFSV (poppet-type design) changes depending on how the control variable of the DCS is modulated. There are five distinct operation modes [70,72]:

1) Deactivated Mode: the pulse duration $\left(t_{ON}\right)$ is so short that the poppet does not move;
2) Ballistic Mode: increasing the pulse duration $\left(t_{ON}\right)$ the poppet starts to move, but it does not reach the upper end stop and is pushed back to the lower end stop;
3) Normal Mode: the pulse duration $\left(t_{ON}\right)$ is long enough to fully open the valve;
4) Inverse Ballistic Mode: the pause time $\left(t_{OFF}\right)$ is so short that the poppet cannot reach the lower end stop;
5) Activated Mode: the pause time $\left(t_{OFF}\right)$ is so short that the valve remains always open.

**Fig. 10:**
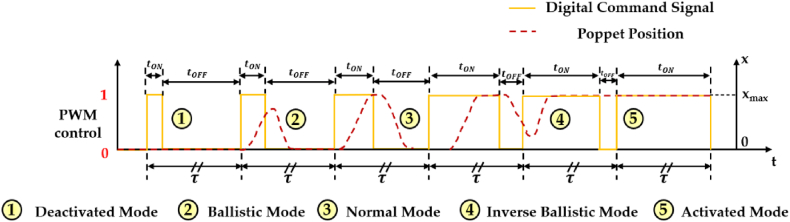
Different behavior of an HFSV controlled by PWM technique, adapted from Ref. [70].

Fig. 10 depicts the five operation modes of an HFSV controlled by the PWM technique [70]. In the graph, the poppet position of the HFSV is represented by the symbol (x) on the right y-axis.

**Fig. 11:**
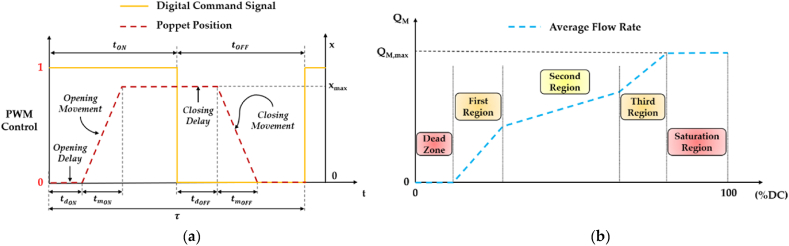
Performance Indicators of an HFSV: (**a**) Schematic Dynamic Movement; (**b**) Static Flow Curve, adapted from Ref. [75].

A thorough examination of Fig. 9, Fig. 10 clearly reveals that the crucial performance indicators of an HFSV encompass its static flow characteristics, dynamic characteristics, power consumption and noise and vibrations [74]. The dynamic characteristic of an HFSV represents its behaviour during opening and closing, while the static flow characteristic depicts the relationship between the average output flow rate and the duty cycle of the pulse control signal, measuring the level of linearity between them [75]. To gain a better understanding of these two performance indicators of HFSVs, Fig. 11 presents the schematic dynamic diagram and the static flow curve of an HFSV (poppet-type design) controlled using the PWM approach.

Fig. 11(a) illustrates the dynamic performance of the HFSV during its operation. This performance is quantified using four key parameters, i.e. the delay before it starts to open (opening delay time, $t_{d_{ON}}$), the duration it takes to fully open (opening moving time, $t_{m_{ON}}$), the delay before it starts to close (closing delay time, $t_{d_{OFF}}$), and the duration it takes to fully close (closing moving time, $t_{m_{OFF}}$). The combined total of the opening delay time and opening moving time defines the opening switching time of the valve, while the combined total of the closing delay time and closing moving time defines the closing switching time of the valve. An important detail to note is the presence of hysteresis in the movement of the HFSV's poppet. The latter is caused by two factors, namely the electrical hysteresis due to the inductance of the coil, and the inherent mechanical hysteresis [76]. These factors result in a delay in the opening switching time when the coil is energized, and a delay in the closing switching time when the coil is de-energized. In contrast, Fig. 11(b) presents the five distinct regions of the static flow curve of the HFSV [77], each corresponding to the operational modes seen earlier in Fig. 10. Examining Fig. 11 closely suggests that longer opening and closing delay times increase the dead zone and saturation region, which in turn decreases the linearity of the flow rate [75]. Additionally, extended opening and closing movement times expand the first and third regions, further reducing linearity [75]. This brief analysis suggests that improving the dynamic performance of the HFSV is crucial for enhancing the linearity of its output flow rate. This improvement can be achieved by reducing the interval time associated with the opening delay, opening movement, closing delay, and closing movement.

**Fig. 12:**
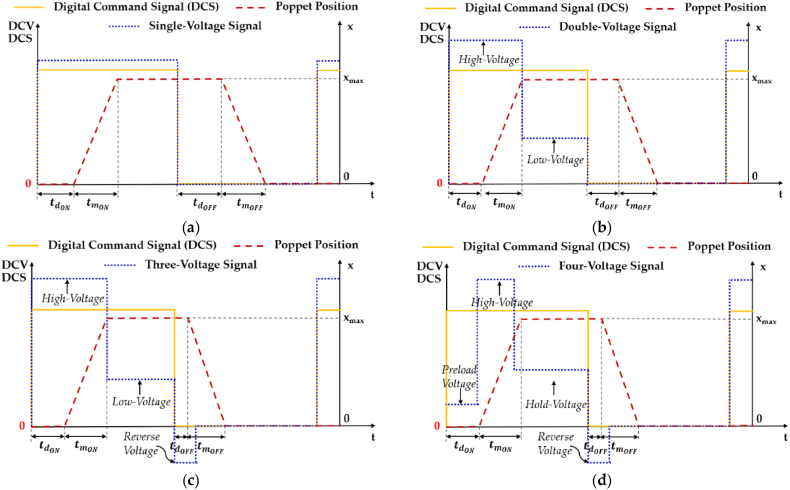
Four different DCV signals used to control a single HFSV: (**a**) Single-Voltage control; (**b**) Double-Voltage control; (**c**) Three-Voltage control; (**d**) Four-Voltage control, adapted from Ref. [75].

To meet the requirements of improved dynamic performance and flow linearity, and reduced power consumption, noise and vibration for a single HFSV, scholars have explored different Discrete Control Voltage (DCV) strategies or various PWM strategies [75]. Regarding the former, the controller adjusts the DCS based on the DCV strategy employed, combining multiple DCV signals with different amplitudes according to a specific logic. Specifically, four DCV signals have been explored over the years. The working principle of each DCV signal is detailed in Table 4, while Fig. 12 provides an illustration for a better understanding [75]. Specifically, Fig. 12 shows how the controller modifies the DCS waveform based on the employed DCV strategy in order to obtain the desired DCV signal.

**Table 4:** Working Principle of four different DCV Strategies [75].

DCV Strategy	Controller Action at a Specific Instant in Time				
	System Start	Rising Edge of DCS	HFSV Critical Max Opening Current	HFSV Maximum Opening	Falling Edge of DCS
Single-Voltage Control	─	DCV with fixed amplitude for valve opening;	─	─	The DCV drops to zero for valve closing;
Double-Voltage Control	─	High voltage for accelerating valve opening;	─	The controller switches to low voltage;	The voltage drops to zero, initiating the valve closing;
Three-Voltage Control	─	High voltage for improving the opening speed;	─	The controller switches to low voltage;	Reverse voltage for increasing closing speed;
Four-Voltage Control	Preload voltage at system start;	High voltage for accelerating valve opening;	The controller switches to holding voltage;	─	Reverse voltage for increasing closing speed;

In single-voltage control, recent research highlights its shortcomings in meeting HFSV requirements for dynamic performance, power efficiency, vibration reduction, and noise control [78]. Double-voltage control, in comparison, enhances both the dynamic performance (in terms of the opening and closing speed) and power consumption, even though it suffers in terms of robustness due to the significant variation in the dynamic characteristics of the HFSV at different oil supply pressures [79]. Three-voltage control innovatively employs reverse voltage during valve closing, rapidly reducing electromagnetic force and improving closing speed, while also improving energy conversion efficiency and robustness by adjusting the DCV signal amplitude [[80], [81], [82], [83]]. Four-voltage control introduces a preload-voltage excitation before valve opening, significantly enhancing dynamic performance (in terms of the opening speed) compared to single-voltage, double-voltage, and three-voltage control strategies [84,85].

On the other hand, in the realm of pulse control strategies, researchers have explored several PWM subcategories, including the compound PWM control, the adaptive PWM control, the intelligent PWM control, and the soft-landing PWM control. The compound PWM control involves multiple pulse control signals with varying carrier frequencies, enhancing the dynamic performance and energy efficiency of HFSVs [86]. The adaptive PWM control adjusts duty cycles of pulse control signals based on oil supply pressure, ensuring optimal dynamic performance under different operating conditions [87]. The intelligent PWM control allows the dynamic characteristics of a single HFSV to be self-adjusted and improved by controlling opening and closing initial currents through intelligent pulse control signals [88,89]. The soft-landing PWM control minimizes noise and vibration during rapid HFSV operations, though it comes with a slight decline in dynamic performance compared to conventional PWM control [90].

To summarize, Table 5 offers a comparison of different control strategies explored for a single HFSV, encompassing both DCV and PWM approaches. The assessment considers aspects such as opening and closing dynamics, energy efficiency, vibration and noise reduction, and robustness. Performance indicators in the table are rated on a scale from 1 to 5, with higher values indicating better performance in the respective category [75].

**Table 5:** PWM and DCV strategies performance comparison for a single HFSV [75].

	Digital Control Approach	Opening Dynamic Performance	Closing Dynamic Performance	Energy Efficient	Noise and Vibration Reduction	Robustness
DCV Strategies	Single-Voltage Control	4	1	1	1	1
	Double-Voltage Control	4	2	4	1	2
	Three-Voltage Control	4	4	3	2	3
	Four-Voltage Control	5	4	2	3	3
PWM Strategies	Compound PWM	4	4	3	1	3
	Adaptive PWM	4	5	4	3	4
	Intelligent PWM	5	5	4	3	4
	Soft-Landing PWM	3	4	3	5	3

Optimal opening dynamics are achieved with the four-voltage control and intelligent PWM control. Regarding closing dynamics, effectiveness is observed in the adaptive PWM control and intelligent PWM control, utilizing a dynamically applied reverse voltage signal. In terms of power consumption, the double-voltage control, adaptive PWM control and Intelligent PWM control are the most efficient, while the single-voltage control is the least efficient. The soft-landing PWM control excels in vibration reduction, and the robustness is highest in the adaptive PWM control and intelligent PWM control, showing minimal sensitivity to variations.

**Fig. 13:**
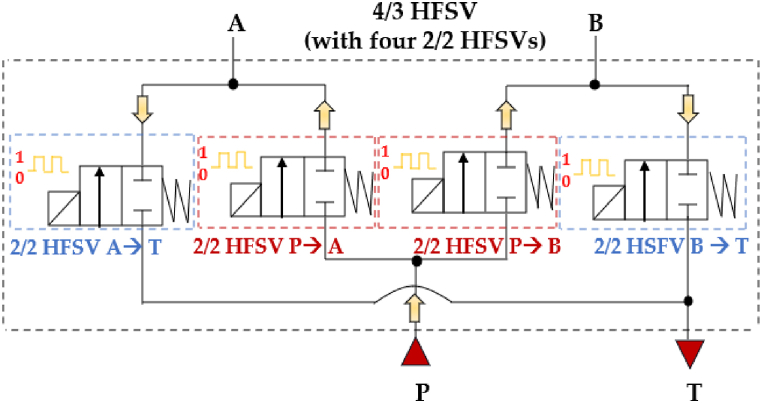
Four-way three-position (4/3) HFSV made up of four 2/2 HFSVs, adapted from Ref. [53].

Independently on the control strategy, HFSVs can be effectively used in a digital hydraulic circuit to replicate the functionality of a conventional four-way three-position (4/3) spool valve. This is achieved by using four 2/2 HFSVs, each responsible for establishing one of the fluid pathways: P → A, A → T, P → B, and B → T. This setup enables the realization of the digital hydraulic circuit of a four-way three-position (4/3) HFSV. The working principle of the 4/3 HFSV, composed of four 2/2 HFSVs, is illustrated in Fig. 13.

**Fig. 14:**
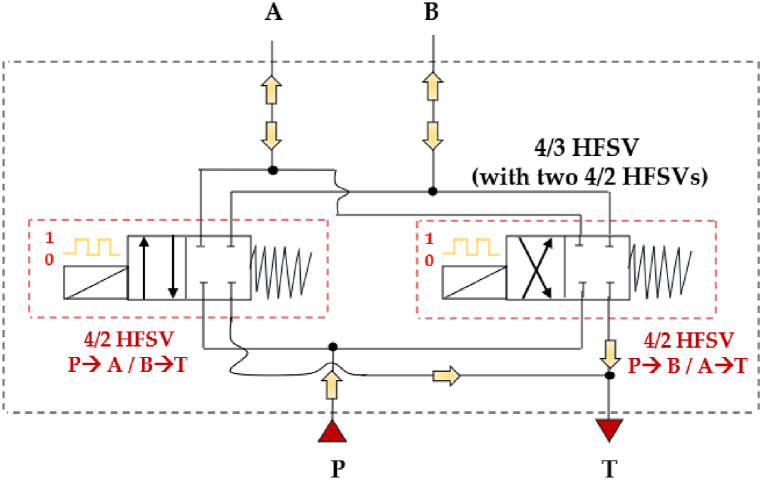
Four-way three-position (4/3) HSFV composed of two 4/2 HFSVs [91].

Interestingly, the system can be simplified by reducing the number of components from four 2/2 HFSVs to just two 4/2 HFSVs. This is achievable if the digital hydraulic circuit of the 4/3 HFSV is configured as shown in Fig. 14 [91].

### 3.2 Parallel digital hydraulic valves

The Parallel Digital Hydraulic Valve, also known as the Digital Flow Control Unit (DFCU), is made up of multiple robust On/Off valves connected in parallel, enabling precise flow control [64]. Each individual On/Off valve in the system possesses key parameter characteristics, including the switching time, reliability, repeatability, and flow capacity [33]. Concerning the latter, there are three conventional control approaches for determining the flow capacity of each On/Off valve within a DFCU [33]:

1. Pulse Number Modulation (PNM): With this approach, the On/Off valves have the same size and, thus, the same flow capacity (1:1:1:1 …);
2. Pulse Code Modulation (PCM): This method is based on the use of the orifices positioned after the valves, which enable the flow capacities of the different valves to be set according to a binary series (1:2:4:8:16 …);
3. Fibonacci Numbers (FN): With this scheme, the orifices positioned after the valves allow the flow capacities of the different valves to be set according to a Fibonacci series (1:1:2:3:5:8:13 …).

Independently on the coding, a DFCU has 2^N^ possible combinations (where N represents the number of parallel connected switching valves), which are called states of the DFCU. By activating and deactivating these valves, the flows from each valve are combined, obtaining different state of the DFCU. This control strategy was already proposed by Flugge-Lotz and Taylor in the past century, where multiple, parallel-operated actuators with different gains could be switched in different logic combinations [92].

Another fundamental property of the DFCU is that it does not require the switching of any individual valve between “on” and “off” to achieve continuous system output. Valve switching is only needed when the state of the DFCU changes [93].

**Fig. 15:**
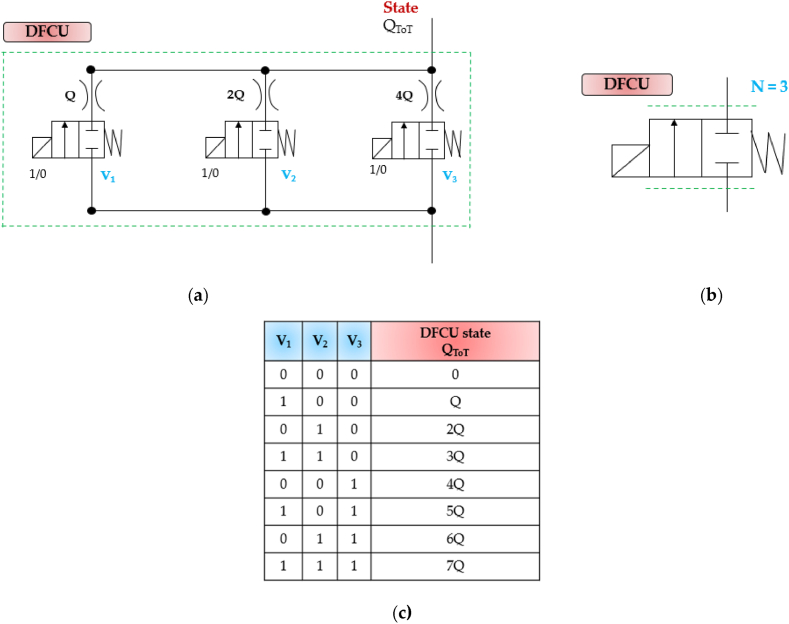
A Digital Flow Control Unit (DFCU) made up of three two-way two-position (2/2) On/Off Valves: (**a**) Complete representation; (**b**) Simplified symbol (N represents the number of parallel connected switching valves); (**c**) Binary state table, adapted from Ref. [48].

Fig. 15 depicts a picture of a DFCU composed of three two-way two-position (2/2) On/Off Valves, along with its simplified symbol and the corresponding working principle using the PCM coding. The two broken lines in the simplified symbol indicate almost proportional flow control of the unit, while the number of valves in the unit is determined by the variable N

**Fig. 16:**
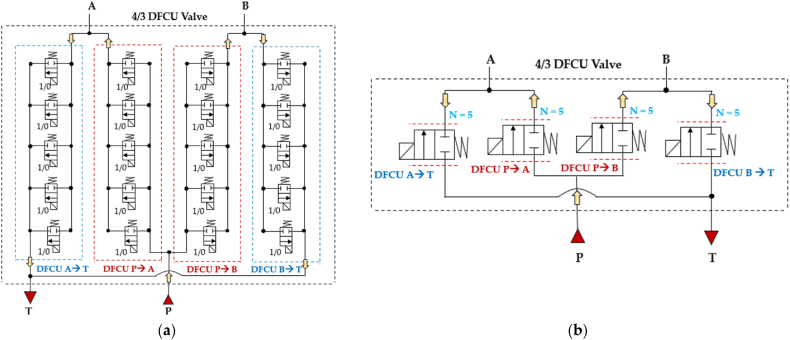
Four-way three-position (4/3) DFCU Valve: (**a**) Complete representation; (**b**) Simplified representation, adapted from Refs. [94,95].

Similarly to the 4/3 HFSV, four independent DFCUs can be used in a digital hydraulic circuit to replace the pathways of a conventional four-way three-position (4/3) spool valve, specifically (P → A, A →T, P → B, B → T), and replicate its function. The working principle of the four-way three-position (4/3) DFCU Valve is shown in Fig. 16, where each of the four DFCUs is composed of five 2/2 On/Off valves [94,95].

**Fig. 17:**
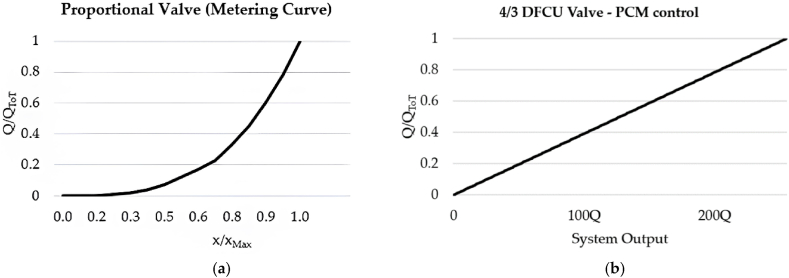
Comparison between analogue and digital hydraulic valves: **a**) Proportional Valve; **b**) 4/3 DFCU Valve, adapted from Ref. [48].

As shown in Fig. 16(a), the On/Off valve that make ups each DFCU is a normally closed two-way two-position (2/2) On/Off valve, which blocks flow when it is in the unactuated position (0) and allows the passage of flow when actuated (1). Clearly, the more precise flow rate is required, the more On/Off valves need to be used in each DFCU [48]. In particular, the same controllability of a conventional spool valve can be achieved if the maximum discrete flow rate provided by each DFCU is approximately 200Q, where Q represents the flow rate provided by the minimum valve of each DFCU, as previously shown in Fig. 15(a). This can be obtained if each DFCU is equipped with 200 equally sized On/Off valves (using PNM coding), 11 On/Off valves (with FN coding) or 8 On/Off valves (via PCM coding) [33]. Fig. 17 illustrates a performance comparison between a 4/3 proportional valve, which uses machined notches and grooves on the sliding spool to achieve the desired flow rate trend based on spool position [12], and a 4/3 DFCU Valve consisting of four DFCUs, each incorporating 8 On/Off valves and controlled through PCM coding [48]. In the graphs of Fig. 17, x/x_Max_ represents the ratio between the spool stroke and the maximum spool stroke; whereas Q/Q_ToT_ is the normalized flow rate, namely the ratio between the flow rate and the maximum flow rate provided by the valve.

Regarding the coding schemes, the Pulse Code Modulation (PCM) is the most used for controlling DFCUs [96]. Its main advantage is the high resolution, namely the ratio between the maximum flowrate and the largest step between two consecutive flow-steps [97]. However, the PCM scheme has an important drawback, namely the high probability of pressure peaks caused by non-uniform On/Off valve switching times [98]. During DFCU state changes, some valves may close before others open, resulting in brief periods of undesired flow rates, which led to pressure peaks [99]. Conventional solutions such as accumulators, pressure relief valves, and orifices are often expensive and can negatively affect system dynamics [100]. In response, scientific researchers have proposed other solutions, such as altering the holding time of the valves [101], or synchronizing their switching time [102,103]. Additionally, modifying the coding scheme is another option for mitigating the pressure peaks [102,104].

**Fig. 18:**
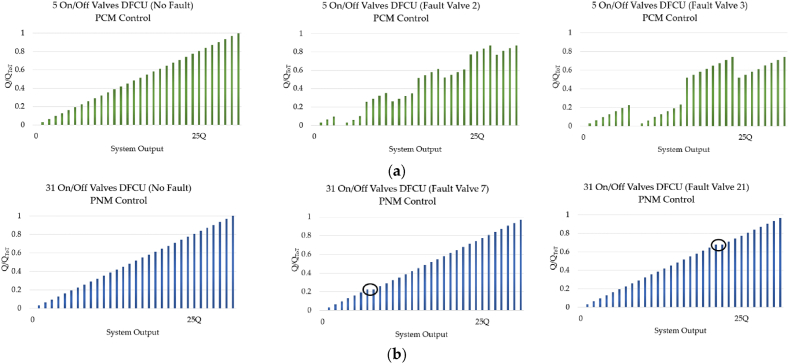
Fault-tolerance performance: (**a**) PCM coding; (**b**) PNM coding, adapted from Ref. [53].

The Pulse Number Modulation (PNM) is the simplest control approach that uses On/Off valves with the same size and, thus, with equal flow capacities. The PNM scheme is considered theoretically superior to the PCM coding due to its faster speed, lower power consumption, and excellent fault-tolerance performance [105,106]. Furthermore, it is also considered the most effective strategy for addressing pressure peak issues. This is because, when the DFCU state changes, the valves, being of the same size, open and close simultaneously [99]. Fig. 18 shows a comparison of the fault-tolerance performance between the PNM and the PCM approaches used to control a DFCU [53].

**Fig. 19:**
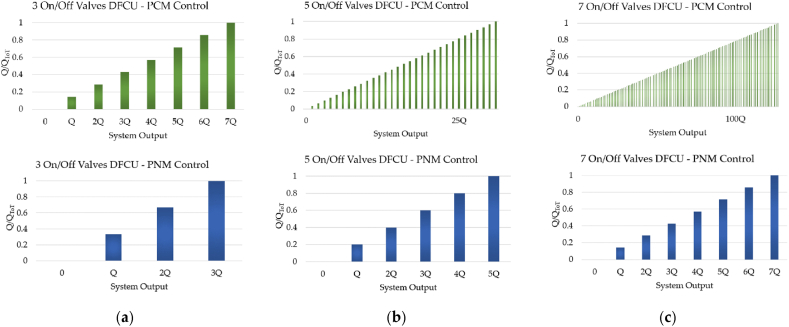
Comparison between the PCM and the PNM control approaches: (**a**) DFCU made up of 3 On/Off Valves; (**b**) DFCU made up of 5 On/Off Valves; (**c**) DCU made up of 7 On/Off Valves, adapted from Ref. [53].

The main weakness of the PNM coding scheme is due to the fact that a large number of On/Off valves is needed in order to obtain a good resolution. Unfortunately, considering the size problem of a DFCU, the PNM control approach cannot be used in most situations [53]. Fig. 19 shows the poor resolution of the PNM control approach compared to the PCM coding scheme.

As shown in Fig. 19(c), a DFCU with 7 On/Off valves achieves a maximum flow rate of 128Q, when the PCM coding is involved. In contrast, the DFCU offers a maximum flow rate of only 7Q, when the PNM is used.

The Fibonacci Numbers (FN) control approach is a compromise between the PCM and the PNM coding. It has the advantage of limiting pressure peaks more effectively than binary coding while requiring fewer valves than the PNM approach [99]. Additionally, it enhances redundancy and enables optimization of valve operations [33].

The strengths and weaknesses identified in conventional DFCU coding schemes underscore the persistent challenge in formulating an effective control strategy that shows high-performance in various aspects, encompassing control accuracy, valve lifespan, and pressure peak management. Consequently, researchers have recently shifted their focus towards investigating the potential application of PWM approaches for DFCU control [75]. This technique uses PWM signals with different duty cycles to individually control each On/Off valve that is connected in parallel within a DFCU, enabling accurate flow rate adjustments [107]. However, this method presents several challenges, such as pressure peaks, a shorter lifespan, and the production of vibration noise [75].

In an effort to address these challenges, a combination of different coding control strategies, such as PWM and PCM has been explored [51]. This combined strategy, compared to the PCM coding scheme, not only provides superior control performance but also helps to mitigate the impact on the flow rate during valve switching [75]. As a result, it presents a promising solution for improving both the efficiency and longevity of DFCUs.

In summary, Table 6 offers a comparison of the performance of control strategies for DFCUs. The performance indicators in the table are represented by values ranging from 1 to 5, where a higher value signifies better performance in the corresponding category [75].

**Table 6:** Control strategies performance comparison for DFCUs [75].

Digital Control Approach	Control Accuracy	Lifespan	Pressure Peaks
PCM	3	3	3
PNM	1	5	1
FN	2	4	2
PWM	5	1	5
PWM + PCM	4	2	4

Table 6 indicates that the PWM approach stands out in terms of control accuracy. However, it does not perform as well when considering DFCUs’ lifespan and pressure peaks problem. In these aspects, the PNM scheme is shown to be the most effective among all coding schemes. The table also highlights that PCM coding is the most commonly used strategy due to its balanced performance across all categories.

## 4 Research Advancements in Digital Hydraulic Valves

Digital hydraulic valves are the key components of digital hydraulic technology. Considering their benefits over conventional analogue spool valves, this section focuses on exploring the research progress and performance of digital hydraulic valves developed over the years.

### 4.1 Research progress in High frequency switching digital hydraulic valves

HFSVs are designed to meet specific criteria. These include the ability to achieve high switching frequencies and high switching speeds, specifically less than 5 ms. Additionally, HFSVs aim to minimize pressure losses and deliver a large flow rate while maintaining a compact size [53]. Currently, the fastest valves can achieve switching frequencies ranging from 50 to 150 Hz, with the potential to reach up to 1000 Hz for short-term operation in very small valves [108].

In literature, the various high frequency switching digital hydraulic valves can be classified into two main categories based on their actuation system:

• HFSVs with electromagnetic actuators;
• HFSVs with smart materials.

#### 4.1.1 HFSVs with electromagnetic actuators

Research publications on the development of HFSVs with electromagnetic actuators can be found regularly from 1970s [[109], [110], [111], [112]]. All these valves presented very fast response times, but their flow rates were limited to very low flow applications. A valve capable of producing higher flow rates was realized in Ref. [113]. However, it presented a complex design and high energy consumption.

**Fig. 20:**
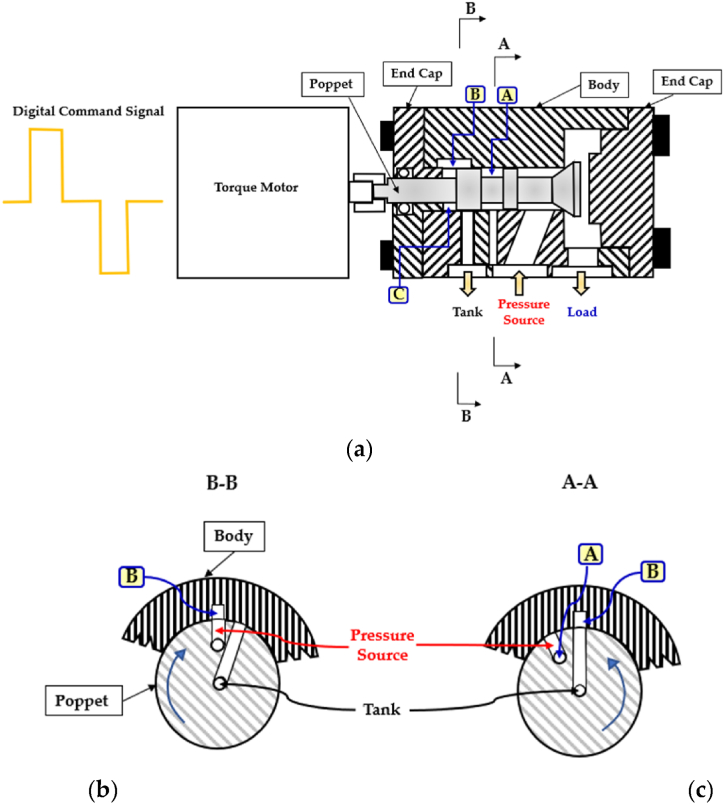
Redrawn schematic representation of the 2/2 HFSVs realized in Ref. [114]: (**a**) Basic Configuration; (**b**) Operating Principle to Open the Valve; (**c**) Operating Principle to Close the Valve.

In 1991, to increase the flow rate, minimize the power consumption, and at the same time, maintain simplicity of design, Cui et al. designed a novel rotary 2/2 HFSV (poppet type) [114]. It presented a single stage structure but could perform as a double-stage valve. Fig. 20 provides an illustration of the operational principle. Referring to Fig. 20(a), the pressurized fluid initially entered chamber (A) which was sealed, causing no movement of the poppet. A DCS was then delivered to the torque motor, allowing the poppet to rotate by a small angle in the position shown in Fig. 20(b). This action aligned the machined ports on the poppet with the ports on the valve body, thereby allowing the pressurized fluid to flow through the path (B) and reach chamber (C). The pressure imbalance between the supply and load pressure caused the poppet to move to the right, opening the valve. To close the valve, the torque motor rotated the poppet counterclockwise, as illustrated in Fig. 20(c). In this way, the chamber (C) was connected with the tank, and the pressure difference between the supply and tank pressures caused the poppet to return to its original position, closing the valve.

To assess the validity of the valve design, a prototype was fabricated, and experimental tests were conducted. The results showed that with a pulse signal frequency of 50 Hz, a duty cycle of 80%, and a pressure drop of 90 bar, the valve had a switching time of approximately 2.5 ms and produced a flow rate of around 18 L/min. However, the value of the flow rate decreased to 10 L/min when the pressure drop was set to 50 bar.

**Fig. 21:**
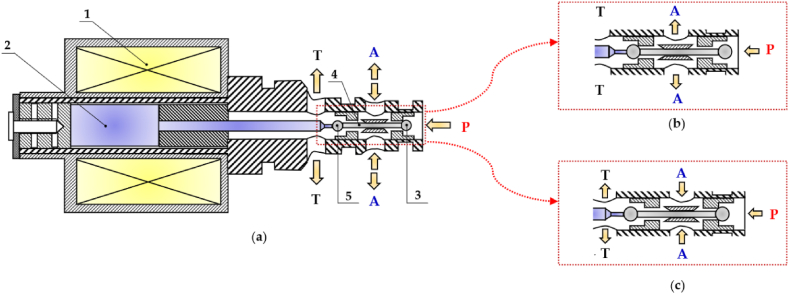
Redrawn schematic representation of the 3/2 HFSV realized in Ref. [115]: (**a**) Basic Configuration: (**b**) Open Position; (**c**) Closed Position.

Three years later, the Chinese company Guizhou Honglin Machinery developed a 3/2 HFSV (ball type), whose schematic representation is shown in Fig. 21(a) [63,115]. When the coil (1) was energized, the electromagnetic force caused the armature (2) and the entire ball assembly (i.e., supply ball (3), separating pin (4), and returning ball (5)) to move to the right. As a result, the port (A) was connected to port (P) and separated from port (T), as depicted in Fig. 21(b). Conversely, when the coil was de-energized, the hydraulic pressure of the inlet port (P) pushed back the ball assembly to the left side. As a result, the port (A) was connected to port (T) and separated from port (P), as illustrated in Fig. 21(c).

According to manufacturer specifications, the opening and closing switching times of the valve were about 3.5 ms and 2.5 ms, respectively [63]. The maximum flow rate of 9 L/min was obtained at a pressure drop of 200 bar [63]. In recent years, this valve has been the subject of several scientific publications. Kong et al. enhanced the dynamic response of the 3/2 HFSV by replacing the single coil with multiple parallel solenoids [116]. By applying a driving voltage of 24 V at a pressure drop of 150 bar, the valve switching times were reduced from 1.5 ms to 1.16 ms for opening and from 1.54 ms to 1.3 ms for closing, respectively. However, the use of a parallel configuration also resulted in increased energy consumption and temperature of the valve. To minimize the energy consumption and temperature rise of the 3/2 HFSV, Zhong et al. proposed an intelligent PWM algorithm for controlling the valve in Ref. [88]. The experiment results showed that the algorithm had the potential to make the valve switching times faster, with the opening time being reduced by 23.6% and the closing time by 17%. Moreover, it significantly decreased energy consumption by 88.8% and limited the temperature rise by 69.9%.

**Fig. 22:**
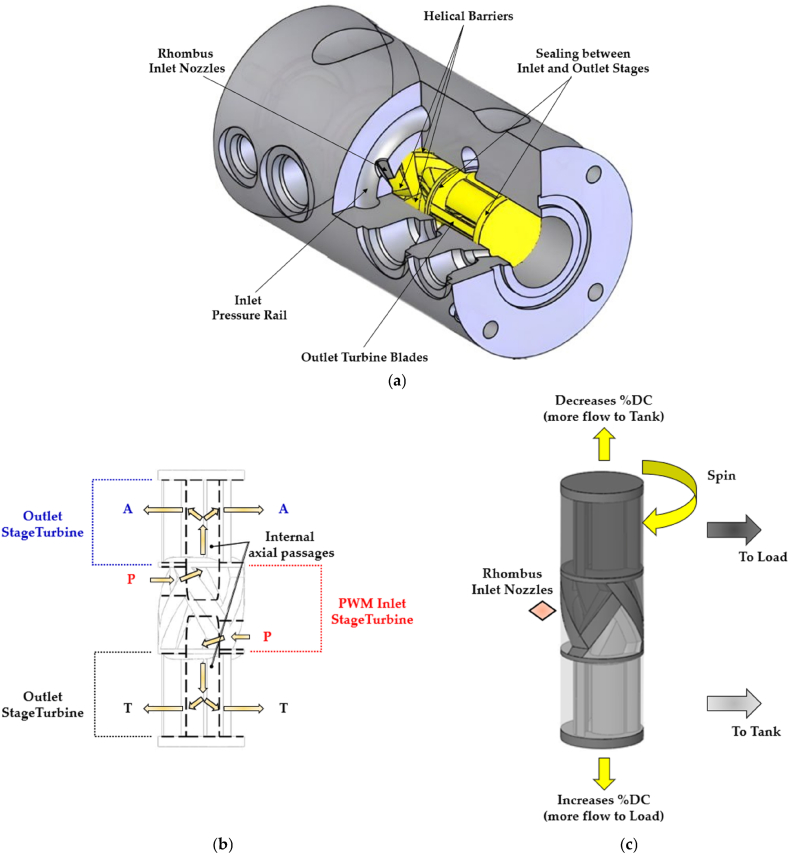
Redrawn schematic representation of the self-spinning rotary 3/2 HFSV proposed in Ref. [117]: (**a**) Cutaway illustration of spool and sleeve assembly; (**b**) Internal geometry of the spool; (**c**) Schematic representation of the rotary spool.

In 2007, Tu et al. proposed a self-spinning rotary 3/2 HSFV (spool type) [117]. The operating principle of this rotary valve can be described by examining Fig. 22. In particular, considering the illustration in Fig. 22(a), the inlet pressure rail on the valve sleeve supplied the rhombus inlet nozzles for generating fluid momentum. The helical barriers captured the fluid momentum and redirected it towards the centre of the valve spool. After reaching the valve spool centre, the fluid was compelled to move in the axial direction via the internal axial passages that guided it to the outlet turbines, as shown in Fig. 22(b). Then, the outlet turbine blades re-accelerated the fluid outward and tangential to the spool. As a result, a torque was generated on the spool, enabling its rotation. With the spool rotation, the inlet turbine continuously switched the fluid flow between the load and the tank, as illustrated in Fig. 22(c.) The duty cycle of the DCS was regulated based on the spool axial position, which modulated the orifice area ratio between the two hydraulic pathways during each rotation. To study the performance of this valve configuration, a detailed dynamic model was realized in Matlab. The numerical predictions showed that the valve could achieved a switching frequency of 84 Hz, and it could produce a flow rate of 40 L/min at a pressure drop of 70 bar.

**Fig. 23:**
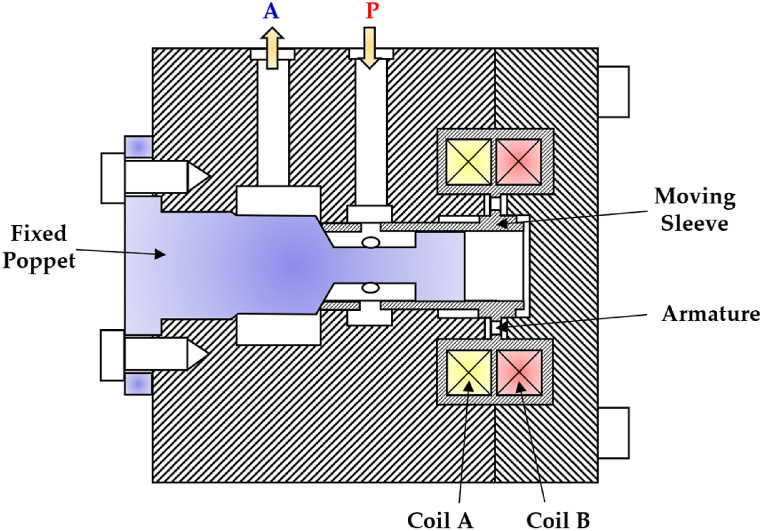
Redrawn schematic representation of the 2/2 HFSV realized in Ref. [118].

In 2013, Gu et al. developed a 2/2 HFSV with a fixed poppet and a moving sleeve, as shown in Fig. 23 [118]. In the closed position, the Coil A was typically energized to counteract the flow force directed towards the right caused by the high inlet pressure from port (P). On the other hand, energizing the Coil B allowed the valve to open by moving the thin moving sleeve towards the right, thereby connecting the high-pressure port (P) to the outlet port (A).

The tested valve prototype achieved an opening switching time of 2.25 ms and a closing switching time of 2.15 ms. Additionally, at the maximum duty cycle of the DCS and a switching frequency of 50 Hz, it delivered a flow rate of approximately 64 L/min with a pressure drop of 10 bar.

**Fig. 24:**
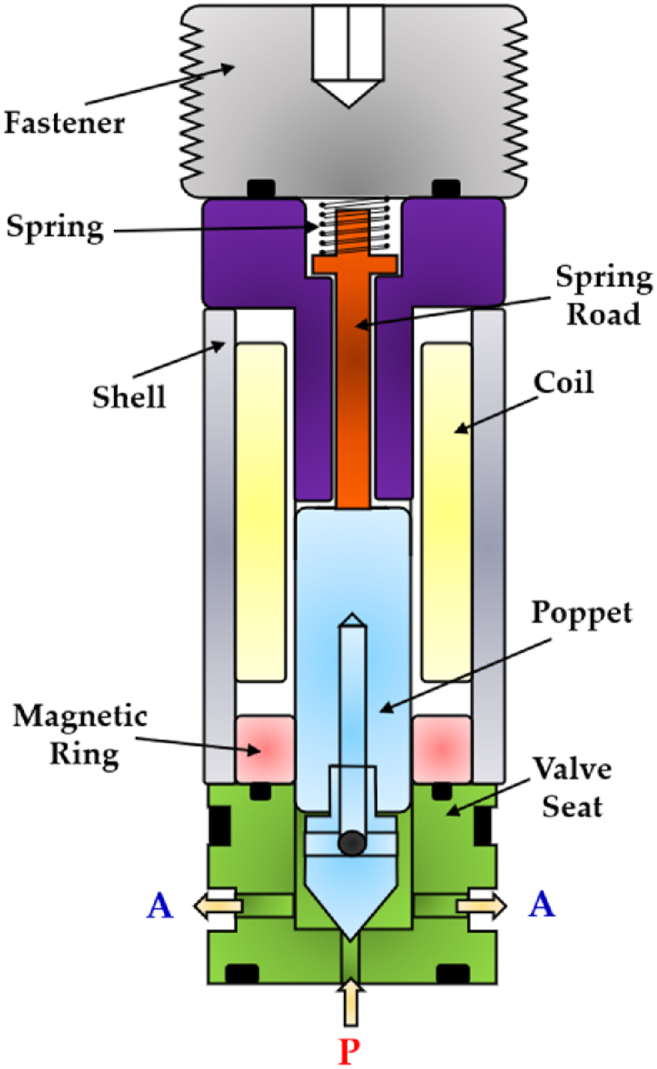
Redrawn schematic representation of the 2/2 HFSV realized in Refs. [[119], [120], [121]].

In 2018, Yang et al. developed a novel miniature 2/2 HFSV (poppet type) [[119], [120], [121]], which is illustrated in Fig. 24. To increase the magnetic flux, the return spring was designed at the top of the valve. This design enabled the production of a significant electromagnetic force despite the valve small size, resulting in excellent response speed. Moreover, the valve structure included a magnetic ring composed of soft magnetic material, which reduced the overall reluctance losses.

The experimental results revealed that the valve exhibited a switching time of 1.5 ms. Nevertheless, due to the small diameter of the inlet orifice (0.5 mm), the flow rate at a pressure drop of 35 bar was limited to only 0.7 L/min.

Table 7 provides a useful comparison of the strengths and weaknesses of the different HFSVs driven by electromagnetic actuators, highlighting the performance of each type.

**Table 7:** Overview of HFSVs actuated by electromagnetic actuators.

Reference	HFSV Type	Description	Switching Frequency [Hz]	Switching Time [ms]	Flow Capacity [L/min] (@ Pressure drop [bar])
Cui et al. [114]	2/2	A rotating poppet allows to control the flow of hydraulic oil	50	2.5	18 (90)
Honglin Machinery [115]	3/2	A ball assembly of three parts enables the flow path to be alternate	–	3.5/2.5 (On/Off)	9 (200)
Tu et al. [117]	3/2	A self-spinning rotary valve continuously switches the flow between the load and the tank	84	–	40 (70)
Gu et al. [118]	2/2	A fixed poppet and a thin moving sleeve cause the valve to open and close	50	2.25/2.15 (On/Off)	64 (10)
Yang et al. [[119], [120], [121]]	2/2	A miniature poppet valve with top spring and soft magnetic ring for low reluctance losses	–	1.5	0.7 (35)

#### 4.1.2 HFSVs with smart materials

The need for high switching speeds led researchers to explore the possibility of using smart materials for actuating HFSVs. In particular, their focus was on piezo-electric actuators. Indeed, the excellent characteristics of these actuators, such as simple design, reduced moving parts, high reliability, and fast response, make them useful for developing this type of digital hydraulic valves [122].

**Fig. 25:**
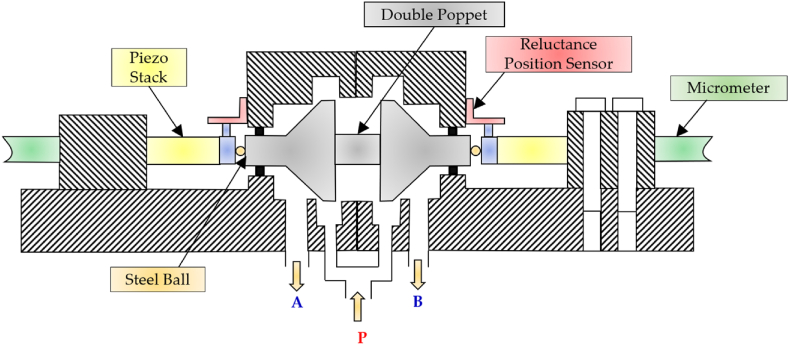
Redrawn schematic representation of the 3/2 HFSV realized in Refs. [123,124].

Back in 1990s, Yokota et al. conducted a research study on the application of piezoelectric actuators to develop a novel 3/2 HFSV (poppet type) [123,124]. A cross-section view of the valve is shown in Fig. 25. There were two piezo-stacks that allowed to move a double poppet through steel balls in a push-pull mode from both sides. The multilayer piezoelectric actuators offered a free stroke of 0.015 mm and a blocking force of 850 N at a maximum operating voltage of 100 V. The displacement of the double poppet was measured by means of a non-contact reluctance-type position sensor. In order to obtain high-speed response, feedforward control was utilized. The authors stated that this valve had a switching frequency of 2 kHz and a switching time of 0.06 ms in both directions, and it could deliver 7.2 L/min at a pressure drop of 100 bar. They recommended the use of this HFSV as a pilot stage for other hydraulic components.

**Fig. 26:**
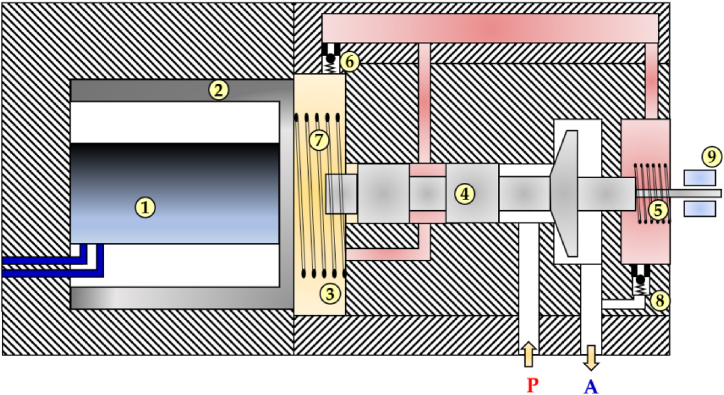
Redrawn schematic representation of the 2/2 HFSV realized in Ref. [125].

To address the issue of high costs associated with the use of two piezo stack actuators, Yamada et al. developed a 2/2 HFSV prototype that employs a spring mechanism and a single piezoelectric actuator to obtain bidirectional poppet control [125]. A cross-section view of the prototype is shown in Fig. 26. Since the main weakness of piezo stack actuators is their low stroke, a hydraulic amplifier was implemented to copy with this problem and actuate the valve. When a DCS was applied to the multilayer piezoelectric actuator (1), the press piston (2) moved towards the right, compressing the oil in the oil chamber (3). This pressurized oil allowed the valve to fully open by pushing the poppet (4) to the right. The poppet movement was countered by a spring (5), which allowed the valve to close when the DCS to the stack was removed. During the closed position, the oil pressure in the oil chamber decreased, and the oil could be recharged through the check valve (6). Other important components were the pre-compression spring (7), the check valve (8), and the LVDT (9). The pre-compression spring (7) ensured the correct value of preload to the piezo stack, while the check valve (8) acted as a pressure relief valve by allowing the red path to remain under a certain value of pressure. The LVDT (9) was used to measure the poppet position and achieve closed loop control.

The experimental results showed that the valve could operate at a frequency of 500 Hz, with a switching time less than 0.7 ms. At a pressure drop of 100 bar and a switching frequency of 100 Hz, the flow rate increased in proportion to the duty cycle until reaching a maximum of 3 L/min at a duty cycle of 75%. However, above this duty cycle value, the flow rate sharply decreased due to the low “off” time of the valve, which did not allow the oil to adequately recharge the oil chamber. As a result, the hydraulic amplification was not enough to open the valve.

**Fig. 27:**
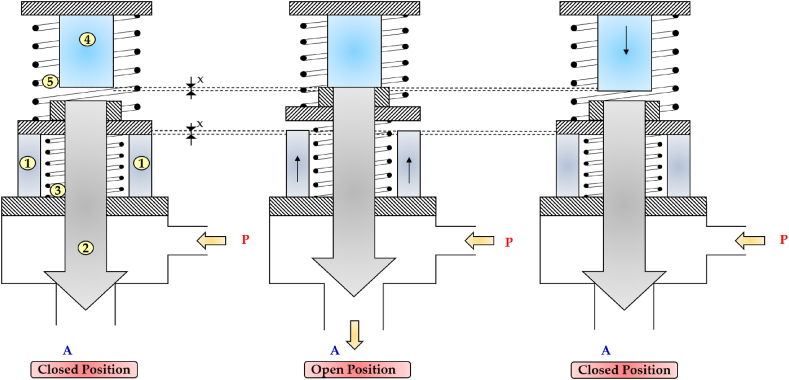
Redrawn working cycle of the 2/2 HFSV proposed in Ref. [126]: Closed Position (left); Open Position (middle); Closed Position (right).

In order to overcome the limitations in duty cycle and achieve higher flows, Ouyang et al. utilized three piezo stack actuators in a 2/2 HSFV to open and close a poppet [126]. The operating principle is depicted in Fig. 27 (from left to right). When both piezo stacks (1) were activated, the combined output force of 3 kN permitted the poppet (2) to initiate a slight opening. At that point, the outlet pressure, $p_{A}$, increased rapidly, while the resistance forces (namely the flow forces and the pressure forces due to the supply pressure, $p_{P}$) decreased quickly. Consequently, the poppet continued to move until it reached its end stop with the help of the spring (3). To return the poppet to its original position, the DCS was removed from the two piezo stacks (1) and applied to piezo stack (4). This latter stack generated an instant force of 3 kN, which impacted the poppet, causing it to move downwards. Subsequently, the force of the spring (5) allowed the poppet to close the valve.

This valve design successfully overcame the issue of micro-displacement of piezo stack actuators. While both piezo stacks (1) and (4) provided a free stroke of 0.032 mm under a maximum voltage of 120 V, the poppet stroke could reach displacements up to 1 mm. To assess the validity of this valve configuration, a detailed mathematical model was realized. The simulations showed that the valve was capable of producing 10 L/min at a pressure drop of 200 bar and a switching frequency of 200 Hz. The graphs also revealed that the switching time of the valve was less than 1 ms.

**Fig. 28:**
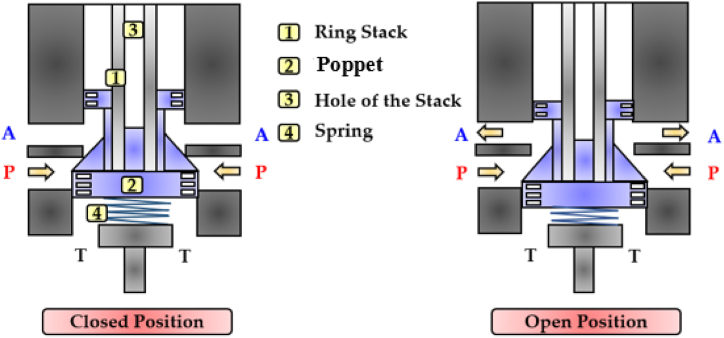
2/2 HFSV proposed in Ref. [31]: Closed Position (left); Open Position (right).

Due to the low flow rate delivered, all the piezoelectric HFSVs analyzed thus far cannot be used to replace the functionality of a conventional spool valve. Therefore, Tamburrano et al. conducted a feasibility study on a novel 2/2 HFSV valve architecture (poppet type) that could be used in the digital hydraulic circuit shown in Fig. 13 [31]. The valve architecture and its working principle are shown in Fig. 28. As the DCS was applied to the ring stack actuator (1), the poppet (2) moved downward, opening the valve. The poppet, which was inserted through the hole of the stack (3), was kept in contact with the piezo actuator by means of a spring (4) which in turn ensured a correct pre-compression to the ring stack.

A comprehensive numerical model, simulating the performance of the valve architecture, was created within the Simulink environment. The numerical predictions showed that, for a switching frequency of 200 Hz, a maximum flow rate of 70 L/min was obtained for a pressure drop of 10 bar and a duty cycle of 100%. Furthermore, the switching time of the valve was within 1 ms.

**Fig. 29:**
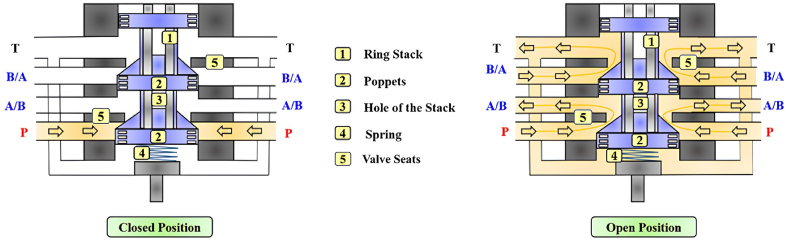
4/2 HFSV proposed in Ref. [91]: Closed Position (left); Open Position (right).

To simplify the digital hydraulic circuit from that shown in Fig. 13 to that presented in Fig. 14, Tamburrano et al. improved upon their previous 2/2 HFSV Simulink model [31] by introducing a novel 4/2 HFSV architecture that utilized the same ring stack actuator [91]. Its operating principle is shown in Fig. 29. Specifically, a DCS triggered the ring stack actuator (1) to initiate valve opening, causing the poppets (3) to move downward and disengage from their valve seats (5). Once again, a spring (4) was employed in the design for the dual purpose of maintaining contact between the poppets and the piezoelectric actuator and ensuring proper pre-compression of the latter.

The open-loop predictions showed that at a duty cycle of 100% and a DCS amplitude of 5 V, with an overall pressure drop of 15 bar, the valve provided an average flow rate of 60 L/min and a power dissipation of only 1500 W. Moreover, the valve's switching time was less than 1 ms.

Table 8 provides an overview of HFSVs actuated by smart materials, highlighting the advantages and disadvantages of the piezoelectric actuation. In contrast to solenoid driven HFSVs, HFSVs utilizing piezoelectric actuators can achieve higher switching frequency and faster switching time. For example, the piezoelectric HFSV developed by Yokota et al. can switch at a frequency of 2 kHz [123,124]. Furthermore, the innovative piezoelectric HFSVs exhibit high flow rates at low pressure drops, as demonstrated in valve designs proposed by Tamburrano et al. [31,91]. However, due to the limited displacement of piezoelectric actuators, additional piezostacks or displacement mechanism amplifiers are often required, as seen in the valve layouts proposed by Ouyang et al. [126] and Yamada et al. [125], which can increase the valve costs.

**Table 8:** Overview of HFSVs actuated by piezoelectric actuators.

Reference	HFSV Type	Description	Switching Frequency [Hz]	Switching Time [ms]	Flow Capacity [L/min] (@ Pressure drop [bar])
Yokota et al. [123,124]	3/2	Two piezo stacks allow to drive a double poppet in a push-pull mode from both sides of the valve	2000	0.06	7.2 (100)
Yamada et al. [125]	2/2	A piezo stack with hydraulic mechanism amplification enables the poppet valve to be actuate	500	<0.7	3 (100)
Ouyang et al. [126]	2/2	Three piezo stacks allow to open and close the poppet valve	200	<1	10 (200)
Tamburrano et al. [31]	2/2	A ring stack directly move the poppet to open and close the valve	200	<1	70 (10)
Tamburrano et al. [91]	4/2	A ring stack enables the poppets in creating fluid pathways within the valve	200	<1	60 (15)

#### 4.1.3 Potential application scenarios of High frequency switching digital hydraulic valves

**Fig. 30:**
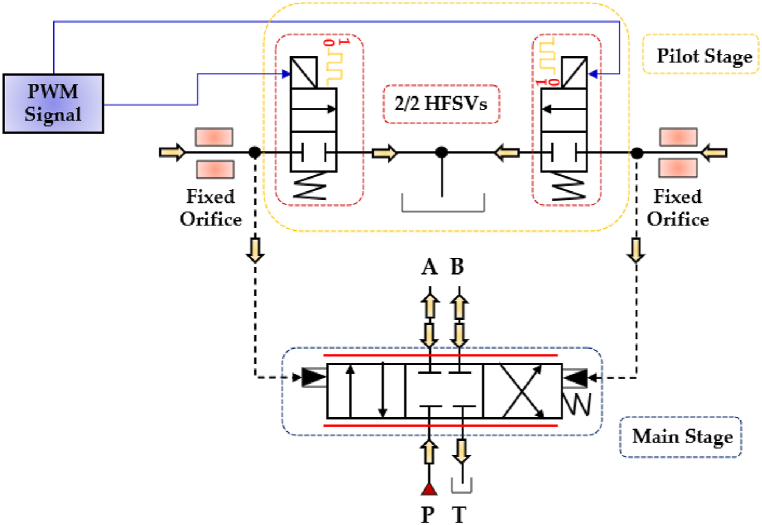
HFSVs as pilot stage in a novel digital servovalve, adapted from Ref. [37].

The previous Table 7, Table 8 have illustrated that both HFSVs equipped with electromagnetic actuators and smart materials are typically associated with small flow rates. As a result, researchers have explored their potential use as control components of other hydraulic components, such as for the main spool of proportional and servovalves [127,128]. A remarkable example is the digital servovalve proposed by Gao et al., which effectively addressed the issues of conventional analogue spool valves, such as oil contamination, high complexity, and low energy efficiency [37]. In this prototype, two 2/2 HFSVs replaced the traditional torque motor structure, as illustrated in Fig. 30. An LDVT was used to measure the position of the main spool and achieve closed loop control. The authors analyzed the effects of control parameters, namely the duty cycle and the switching frequency of the PWM signal and found that increasing the duty cycle improved the main spool displacement, while a higher switching frequency reduced its oscillations. Furthermore, the step test response demonstrated that the main spool could reach the set point in just 3.5 ms. However, due to the hysteresis of the electromagnetic actuators, the switching frequency of the two 2/2 HFSVs was limited to 100 Hz, resulting in reduced position tracking accuracy for the main spool.

**Fig. 31:**
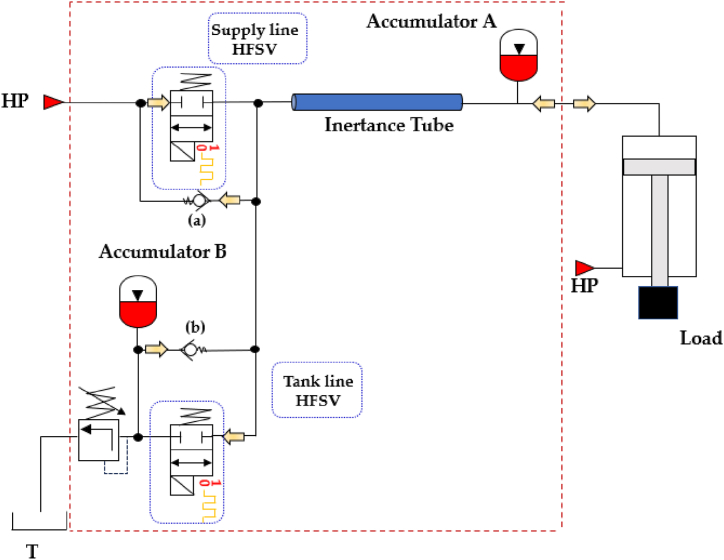
Digital Hydraulic Buck Converters, adapted from Ref. [129].

HFSVs also play a significant role in digital hydraulic buck converters (DHBCs), which, in addition to HFSVs, leverage the reactive behavior of two important hydraulic components, namely accumulators and inertance tubes, as shown in Fig. 31. The capacitive and inertial effect of these hydraulic components, along with the high switching speeds of HFSVs, enables precise adjustment of system pressure and flow rates, energy recovery, and overall system efficiency improvements [129]. Scheidl et al. explored these converters as replacements for conventional spool valves in advanced casting systems, aiming to improve the control of mold oscillations [108]. This implementation, indeed, resulted in reduced energy consumption and lower installation costs. For further details on switched inertance concepts, refer to Refs. [130,131].

**Fig. 32:**
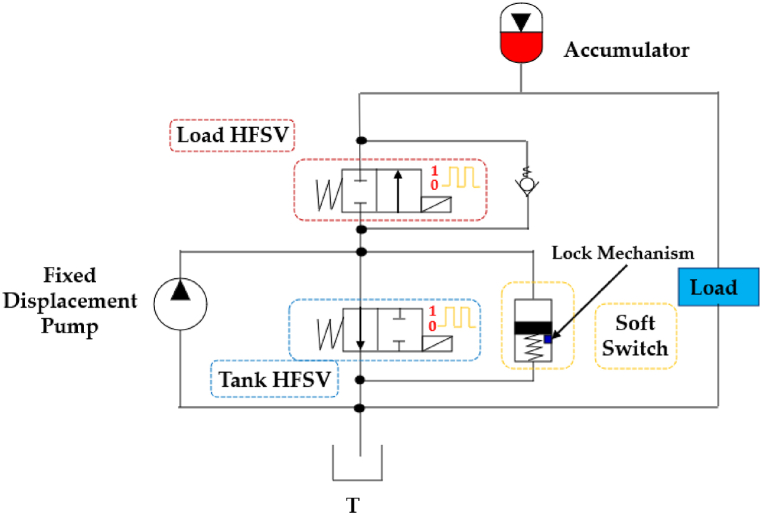
Redrawn of the digital hydraulic system proposed in Ref. [134].

The use of HFSVs is also instrumental in actively and intelligently controlling the displacement, force, and torque delivered by pumps, cylinders, and motors [53]. These digital hydraulic systems have demonstrated their capacity to enhance energy system efficiency, as demonstrated in various scientific publications [132,133]. A notable example is the digital hydraulic system proposed by Rannow and Li [134], which is shown in Fig. 32. In this system two HFSVs, along with a check valve and a soft switch lock-release mechanism, efficiently controlled a hydraulic load, reducing overall system losses by 64% even when the HFSVs operated at a relatively low switching frequency of just 20 Hz.

### 4.2 Research progress in parallel digital hydraulic valves

The idea of using multiple valves in parallel to control the flow of hydraulic fluid date back approximately 100 years ago to the patent of Rickenberg in 1930. He was one of the pioneers to propose the parallel use of three solenoid valves with different flow capacity in his patent application [135]. On the other hand, Bower was the first to use real PCM control with a dual-acting actuator in 1961 [136]. One year later, Murphy and Weil introduced a four-way digital valve that could independently control the load using a DFCU [137]. In 1978, Virvalo demonstrated the application of a DFCU in regulating the velocity of a hydraulic cylinder [138]. However, practical implementation of parallel digital hydraulic valves was challenging at the time due to the limitations of computer technology.

In the new millennium, instead, there has been a substantial increase in research and applications related to parallel digital hydraulic valves. One of the most prominent contributors in this field is the research group led by Matti Linjama at Tampere University of Technology. Their efforts have been focused on developing innovative DFCU structures [139,140] and implementing advanced control algorithms to enhance the overall DFCU performance [[141], [142], [143]].

**Fig. 33:**
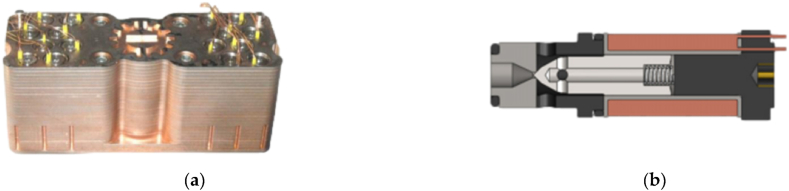
DFCU with sixteen On/Off valves in parallel developed by Paloniitty et al. [144]: (**a**) prototype; (**b**) utilized On/Off valve.

In recent years, advancements have been made in the development of DFCU prototypes that are capable of integrating more switching valves. In 2014, Paloniitty et al. introduced a DFCU containing 16 On/Off valves, as shown in Fig. 33 [144]. The DFCU flow rate was controlled by the PNM coding scheme, and it achieved a flow rate of approximately 25 L/min at a pressure drop of 35 bar, with a switching time of less than 4 ms [145]. To improve the resolution of the flow rate, a novel DFCU was proposed by Linjama et al., in 2015 that integrated 64 On/Off valves [146].

**Fig. 34:**
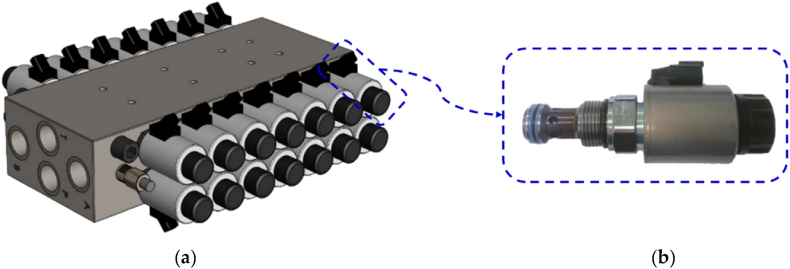
4/3 DFCU Valve with multiple On/Off valves in parallel developed by Ketonen et al. [147]: (**a**) prototype; (**b**) utilized On/Off valve.

Two years later, Ketonen et al. introduced a 4/3 DFCU Valve with high flow characteristics and capable of replacing conventional spool valves in many mobile and industrial applications [147]. As shown in Fig. 34(a), each of the four DFCU consisted of 7 On/Off valves from Bucher Hydraulic and was capable of producing 127 states, resulting in a total of 508 possible flow rate combinations for the entire 4/3 DFCU Valve. The Bucher Hydraulic On/Off valve, illustrated in Fig. 34(b), provided a flow rate of 60 L/min at a pressure drop of 15 bar. The entire prototype presented a dimension of 402x343 × 96 mm. The experimental results showed that, by employing PCM and PNM coding schemes, the measured maximum flow rate of each DFCU could reach up to 200 L/min at a pressure difference of 15 bar. The authors stated that the 4/3 DFCU Valve could offer excellent fault tolerance performance and could be an efficient energy solution to control hydraulic actuators in range up to 400 L/min and pressures up to 350 bar.

#### 4.2.1 Potential application scenarios of parallel digital hydraulic valves

**Fig. 35:**
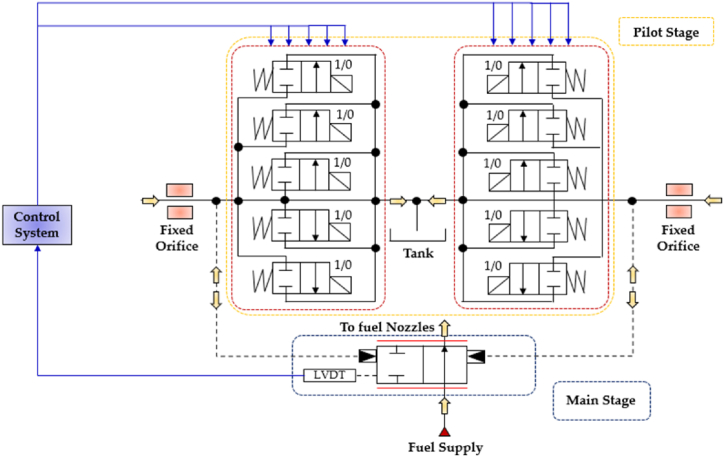
DFCUs as pilot stage in a novel digital servovalve, adapted from Ref. [36].

Recently, in an effort to enhance the performance of electro-hydraulic servovalves used in aircraft fuel systems and address issues associated with these conventional spool valves, Gao et al. replaced the traditional torque motor with two DFCUs to actuate the main spool in a novel digital fuel metering servovalve [36]. As shown in the hydraulic schematic of Fig. 35, each DFCU controlled five parallel-connected On/Off valves through PCM coding scheme. A detailed numerical model was developed to investigate the performance of this valve design and the numerical results revealed that the main spool could reach the desired set point in just 4.1 ms.

**Fig. 36:**
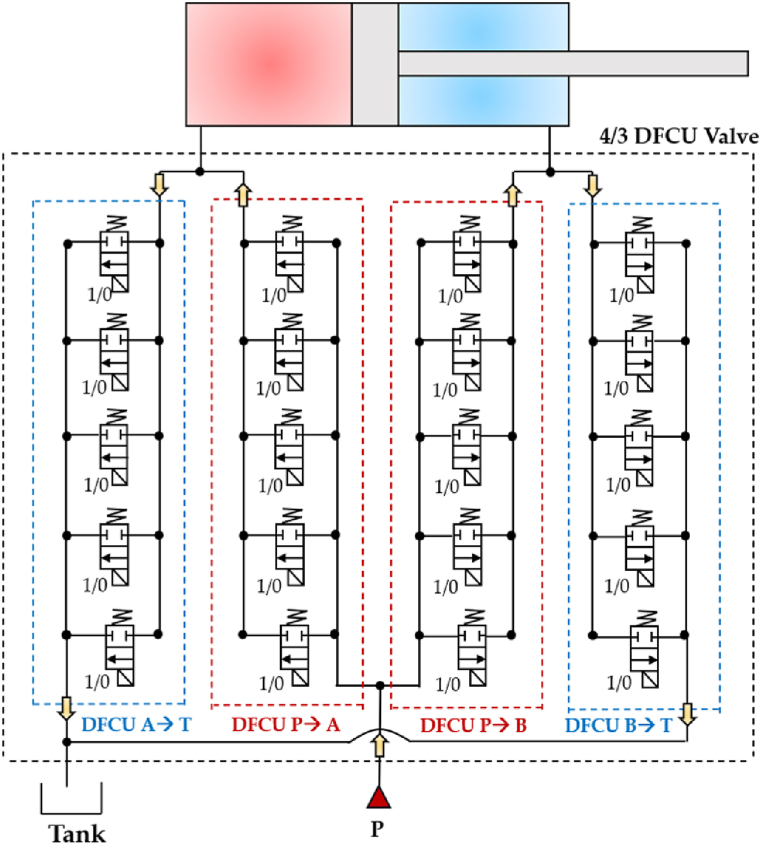
DFCUs used to control independently velocity and pressure level of an actuator, adapted from Ref. [148].

DFCUs can also be used to control independently velocity and pressure level of an actuator. A practical example of this can be seen in the work of Linjama et al., where a 4/3 DFCU was used to regulate the flow rate to a heavily loaded cylinder, as depicted in Fig. 36 [148]. The PCM scheme was employed to control all four flow paths independently, using five on/off valves in parallel for each DFCU, which provided a total of 128 potential flow rate states. This parallel digital hydraulic system, due to its minimal pressure losses, has the potential to be more energy-efficient compared to conventional valve-controlled hydraulic systems that rely on throttling for precise control.

**Fig. 37:**
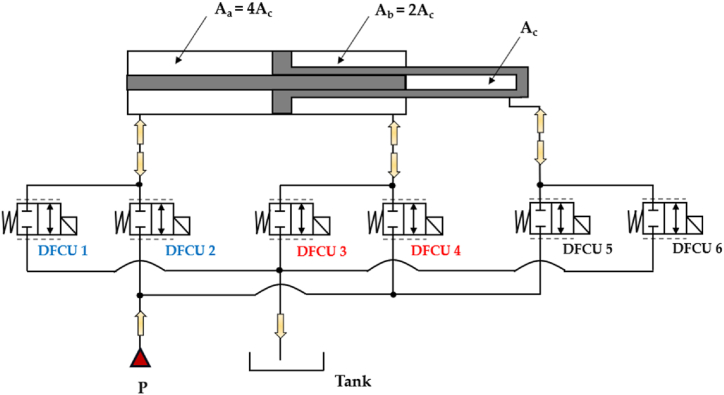
DFCUs used to control a multi-chamber cylinder, adapted form [149].

Following a similar approach, Huova et al. used DFCUs to achieve discrete and intelligent control of a variable displacement linear actuator (or multi-chamber cylinder) [149]. Specifically, the authors employed a pressurized tank line and a constant pressure supply line to effectively drive the three-chamber hydraulic cylinder, as depicted in Fig. 37. To control each chamber, two DFCUs were utilized, with one controlling the flow rate between the chamber and the supply line, and the other controlling the flow rate between the chamber and the tank line. The authors stated that the energy loss in this parallel digital hydraulic system, considering a constant supply pressure, was reduced by 30%–60% compared to conventional hydraulic cylinders. Moreover, multi-chamber cylinders were integrated into construction machinery, resulting in a considerable reduction in fuel consumption [150].

For a more in-depth understanding of the operating principle and displacement control of multi-chamber cylinders, additional information can be found in Refs. [151,152].

## 5 Challenges and Future Directions in Digital Hydraulic Technology

**Fig. 38:**
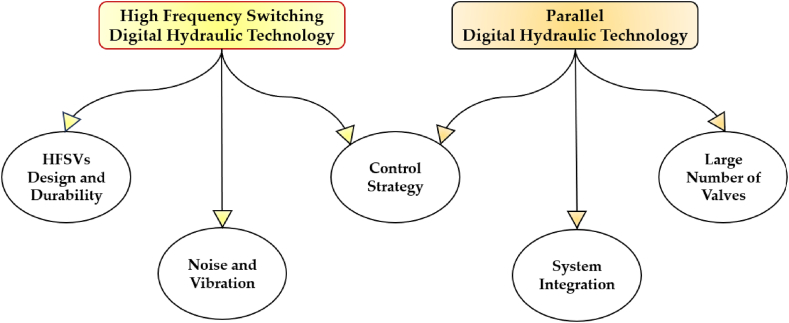
Key challenges in High frequency switching and parallel digital hydraulic systems [153].

Despite the considerable advantages over conventional hydraulic technology, achieving widespread adoption of digital hydraulic technology across diverse industries in the coming years requires addressing several challenges [153]. Fig. 38 illustrates the primary obstacles faced by both parallel and high-frequency switching digital hydraulic technology.

Concerning high-frequency switching digital hydraulic technology, a critical challenge lies in the design and durability of HFSVs. These valves must withstand rapid switching speeds, deliver large flow rates, maintain durability, and resist wear and fatigue. Moreover, the high frequency switching of these components can introduce issues such as noise, vibration, and pressure peaks in the system.

Regarding parallel digital hydraulic technology, the use of a large number of switching valves in DFCUs can result in challenges related to system size, cost, and complexity. Additionally, similar to high-frequency switching digital hydraulic technology, developing and implementing a control strategy can be complex, requiring unconventional approaches compared to conventional hydraulic technology.

Finally, the integration of digital hydraulic technologies into existing machinery poses challenges related to compatibility and space constraints.

## 6 Conclusions

Despite the significant improvements achieved in recent years, conventional analogue spool valves, both proportional and servovalves, continue to experience high energy losses, essentially due to the high-pressure drops encountered by pressurized fluid as it flows across the small narrow passages uncovered during valve control. The outstanding results obtained in information, communication, and power electronics, coupled with the similarities between electrical and hydraulic systems, demonstrate that use of some digital concepts into hydraulic technology could be instrumental to address the energy inefficiency of conventional hydraulic systems. By replacing conventional analogue spool valves with low-cost and robust On/Off valves, digital hydraulic technology aims to minimize power losses and increase the overall energy efficiency of hydraulic systems.

This paper has provided a comprehensive review of the state-of-the-art and research advancements in digital hydraulic technology, with a particular focus on digital hydraulic valves and their control. The digital hydraulic valve architectures developed over the years were thoroughly examined. The performance of each configuration was discussed in detail. The digital hydraulic valves, both DFCUs and HFSVs, have shown that digital hydraulic technology have the potential to revolutionize the field of fluid power in terms of energy consumption reduction.

Although there are still some challenges to overcome, such as the small flow rates of HFSVs, the size problem of DFCUs and the complexity of control strategies, these digital hydraulic valves offer numerous advantages over conventional analogue hydraulic spool valves, including greater robustness and longer lifespan. Therefore, further research and development are crucial to fully realize the potential of this novel technology across various industries in the years ahead.

## Data availability

Sharing research data helps other researchers evaluate your findings, build on your work and to increase trust in your article. We encourage all our authors to make as much of their data publicly available as reasonably possible. Please note that your response to the following questions regarding the public data availability and the reasons for potentially not making data available will be available alongside your article upon publication.

## CRediT authorship contribution statement

**Francesco Sciatti:** Writing – review & editing, Writing – original draft, Visualization, Validation, Supervision, Software, Resources, Project administration, Methodology, Investigation, Funding acquisition, Formal analysis, Data curation, Conceptualization. **Paolo Tamburrano:** Writing – review & editing, Writing – original draft, Visualization, Validation, Supervision, Software, Resources, Project administration, Methodology, Investigation, Funding acquisition, Formal analysis, Data curation, Conceptualization. **Elia Distaso:** Writing – review & editing, Writing – original draft, Visualization, Validation, Supervision, Software, Resources, Project administration, Methodology, Investigation, Funding acquisition, Formal analysis, Data curation, Conceptualization. **Riccardo Amirante:** Writing – review & editing, Writing – original draft, Visualization, Validation, Supervision, Software, Resources, Project administration, Methodology, Investigation, Funding acquisition, Formal analysis, Data curation, Conceptualization.

## Declaration of competing interest

The authors declare that they have no known competing financial interests or personal relationships that could have appeared to influence the work reported in this paper.

## Abbreviations

ABS: Antilock Braking System
DCS: Digital Command Signal
DFCU: Digital Flow Control Unit
DFM: Duty Cycle Frequency Method
DHBC: Digital Hydraulic Buck Converter
DHHA: Digital Hydraulic Hybrid Actuator
DHPMS: Digital Hydraulic Power Management System
DVC: Discrete Voltage Control
FN: Fibonacci Number
HFSV: High Frequency Switching On/Off Valve
ISO: International Organization for Standardization
LVDT: Linear Variable Differential Transformer
PCM: Pulse Code Modulation
PFM: Pulse Frequency Modulation
invPFM: Inverted Pulse Frequency Modulation
PNM: Pulse Number Modulation
PWM: Pulse Width Modulation

## References

[1] Mays L.W. (2008).

[2] Papoutsidakis M., Chatzopoulos A., Papachristos D., Drosos C. (Jun. 2019). Hydraulics and pneumatics: operational characteristics and control for modern industry applications. Int. J. Comput. Appl..

[3] Mokyr J. (2000).

[4] El-Din M.G., Rabi M. (2009). https://www.accessengineeringlibrary.com/content/book/9780071622462.

[5] Moog (2003).

[6] Xu B., Shen J., Liu S., Su Q., Zhang J. (2020). Research and development of electro-hydraulic control valves oriented to industry 4.0: a review. Chin. J. Mech. Eng..

[7] Sciatti F., Tamburrano P., de Palma P., Distaso E., Amirante R. (2022). Journal of Physics: Conference Series.

[8] Tamburrano P. (2022). Journal of Physics: Conference Series.

[9] Sciatti F., Tamburrano P., Distaso E., Amirante R. (2023). Fluid Power Systems Technology.

[10] Tamburrano P., Sciatti F., Distaso E., Di Lorenzo L., Amirante R. (2022). Validation of a simulink model for simulating the two typical controlled ventilation modes of intensive care units mechanical ventilators. Appl. Sci..

[11] Hunt T., Vaughan N. (1996).

[12] Tamburrano P., Plummer A.R., Distaso E., Amirante R. (2019). A review of direct drive proportional electrohydraulic spool valves: industrial state-of-the-art and research advancements. Journal of Dynamic Systems, Measurement and Control, Transactions of the ASME.

[13] Tamburrano P., Plummer A.R., Distaso E., Amirante R. (2019). A review of electro-hydraulic servovalve research and development. Int. J. Fluid Power.

[14] Tamburrano P., Distaso E., Plummer A.R., Sciatti F., de Palma P., Amirante R. (2021).

[15] Amirante R., Moscatelli P.G., Catalano L.A. (2007). Evaluation of the flow forces on a direct (single stage) proportional valve by means of a computational fluid dynamic analysis. Energy Convers. Manag..

[16] Amirante R., Distaso E., Tamburrano P. (2014). Experimental and numerical analysis of cavitation in hydraulic proportional directional valves. Energy Convers. Manag..

[17] Plummer A. (2016). Proc 10th International Fluid Power Conference.

[18] Sun X., Liu S., Bao J., Li J., Liu Z. (2021). A performance prediction method for a high-precision servo valve supported by digital twin assembly-commissioning. Machines.

[19] Gao B., Zhang W., Zheng L., Zhao H. (2023). Research on high-precision position control of valve-controlled cylinders based on variable structure control. Machines.

[20] Heinken H., Ulrich S., Bruns R., Schneider S. (2020). High-response electrorheological servo valve. J. Intell. Mater. Syst. Struct..

[21] Liang N., Yuan Z., Zhang F. (2023). Oil particle-induced erosion wear on the deflector jet servo valve prestage. Aerospace.

[22] Tamburrano P., Plummer A.R., Elliott P., de Palma P., Distaso E., Amirante R. (Dec. 2019). AIP Conference Proceedings.

[23] Tamburrano P. (2020). ASME/BATH 2019 Symposium on Fluid Power and Motion Control, FPMC 2019.

[24] Tamburrano P., Sciatti F., Plummer A.R., Distaso E., de Palma P., Amirante R. (2021). A review of novel architectures of servovalves driven by piezoelectric actuators. Energies.

[25] Ledvoň M., Hružík L., Bureček A., Dýrr F., Polášek T. (2023). Leakage characteristics of proportional directional valve. Processes.

[26] Ellman A. (1998). Proceedings of the ASME 1998 International Mechanical Engineering Congress and Exposition.

[27] Eryilmaz Bora, Wilson Bruce H., B. and B. H. W. Eryilmaz (2000). “Modeling the Internal Leakage of Hydraulic servovalves.” in International Mechanical Engineering Congress and Exposition.

[28] Tamburrano P., Plummer A.R., de Palma P., Distaso E., Amirante R. (2020). A novel servovalve pilot stage actuated by a piezo-electric ring bender: a numerical and experimental analysis. Energies.

[29] Tamburrano P., Plummer A.R., De Palma P., Distaso E., Amirante R. (May 2020). A novel servovalve pilot stage actuated by a piezoelectric ring bender (Part II): design model and full simulation. Energies.

[30] Tamburrano P., Amirante R., Distaso E., Plummer A.R. (2018). Full simulation of a piezoelectric double nozzle flapper pilot valve coupled with a main stage spool valve. Energy Procedia.

[31] Tamburrano P., De Palma P., Plummer A.R., Distaso E., Sciatti F., Amirante R. (2021).

[32] Scheidl R., Linjama M., Schmidt S. (2012). Is the future of fluid power digital?. Proc. IME J. Syst. Control Eng..

[33] Laamanen M.S.A., Vilenius M. (2003). The Eighth Scandinavian International Conference on Fluid Power.

[34] Achten P., Linjama M., Scheidl R., Schmidt S. (2012). Discussion: is the future of fluid power digital?. Proc. IME J. Syst. Control Eng..

[35] Jiao Z., Zhang H., Shang Y., Liu X., Wu S. (2020). A power-by-wire aircraft brake system based on high-speed on-off valves. Aero. Sci. Technol..

[36] Gao Q., Zhu Y., Liu J. (2022). Dynamics modelling and control of a novel fuel metering valve actuated by two binary-coded digital valve arrays. Machines.

[37] Gao Q., Zhu Y., Wu C., Jiang Y. (2021). Development of a novel two-stage proportional valve with a pilot digital flow distribution. Front. Mech. Eng..

[38] Yang M. (2022). Study on the Digital hydraulic driving system of the belt conveyor. Machines.

[39] Brandstetter R., Deubel T., Scheidl R., Winkler B., Zeman K. (2017). “Digital hydraulics and ‘industrie 4.0,’” proceedings of the institution of mechanical engineers, Part I. Journal of Systems and Control Engineering.

[40] Linjama M., Grönholm J., Paloniitty M. (2019). Workshop on Digital Fluid Power.

[41] Rituraj R., Scheidl R. (2022). Fluid Power Systems Technology.

[42] Pan M., Johnston N., Plummer A., Kudzma S., Hillis A. (2014). Theoretical and experimental studies of a switched inertance hydraulic system. Proc. IME J. Syst. Control Eng..

[43] Leifeld R., Vukovic M., Murrenhoff H. (2015). Proceedings of the 7th Workshop on Digital Fluid Power.

[44] Roemer D.B., Norgaard C., Bech M.M., Johansen P. (2016). Proceedings of the Eighth Workshop on Digital Fluid Power.

[45] Noritsugu T. (1986). Development of PWM mode electro-pneumatic servomechanism. I: speed control of a pneumatic cylinder. J. Fluid Control.

[46] Noritsugu T. (1987). Development of PWN mode electro-pneumatic servomechanism. II: position control of a pneumatic cylinder. J. Fluid Control.

[47] Ahn K., Yokota S. (2005). Intelligent switching control of pneumatic actuator using on/off solenoid valves. Mechatronics.

[48] Vemet forward, “https://www.valmet.com/media/articles/up-andrunning/newtechnology/FPDigHydr/.”.

[49] Merrill K., Holland M., Batdorff M., Lumkes J. (2010). Comparative study of digital hydraulics and digital electronics. Int. J. Fluid Power.

[50] Scheidl R., Kogler H., Winkler B. (2013). Hydraulic switching control-objectives, concepts, challenges and potential applications. Hidraulica.

[51] Huova M., Plöckinger A. (2010).

[52] Zhang Q., Kong X., Yu B., Ba K., Jin Z., Kang Y. (2020). Review and development trend of digital hydraulic technology. Appl. Sci..

[53] Linjama M. (2011).

[54] Yang H. (2017). Construction Machinery Technology and Management.

[55] Poncelet J.V. (1874).

[56] Fink C. (1865). Über gebräuchliche Modifikationen des Wattschen Regulators. VDI Zeitschrift.

[57] Farcot J. (1873).

[58] Wang F., Gu L., Chen Y. (2011). A continuously variable hydraulic pressure converter based on high-speed on–off valves. Mechatronics.

[59] Gao Q. (2022). Nonlinear adaptive control with asymmetric pressure difference compensation of a hydraulic pressure servo system using two high speed on/off valves. Machines.

[60] Wang F., Gu L., Chen Y. (2012). A hydraulic pressure-boost system based on high-speed on–off valves. IEEE/ASME transactions on mechatronics.

[61] Ballard R.L. (1968).

[62] Meßner F., Scheidl R. (2016). Proceedings of the Eighth Workshop on Digital Fluid Power.

[63] Wang H., Chen Z., Huang J., Quan L., Zhao B. (2022). Development of high-speed on–off valves and their applications. Chin. J. Mech. Eng..

[64] Laamanen A., Siivonen L., Linjama M., Vilenius M. (2004).

[65] Heikkilä M., Linjama M. (2013). Displacement control of a mobile crane using a digital hydraulic power management system. Mechatronics.

[66] Linjama M., Huova M., Pietola M., Juhala J., Huhtala K. (2015). The Fourteenth Scandinavian International Conference on Fluid Power.

[67] Linjama M., Koskinen K.T., Vilenius M. (2003). Accurate trajectory tracking control of water hydraulic cylinder with non-ideal on/off valves. Int. J. Fluid Power.

[68] Amirante R., Andrea Catalano L., Tamburrano P. (2014). The importance of a full 3D fluid dynamic analysis to evaluate the flow forces in a hydraulic directional proportional valve. Eng. Comput..

[69] Winkler B. (2017). Proc. Of the Ninth Workshop on Digital Fluid Power.

[70] Schepers I., Weiler D., Weber J. (2012). Proceedings of the Fifth Workshop on Digital Fluid Power.

[71] Schepers I., Weiler D., Weber J. (2011). Dynamic Systems and Control Conference.

[72] Schepers I., Schmitz D., Weiler D., Cochoy O., Neumann U. (2011). A novel model for optimized development and application of switching valves in closed loop control. Int. J. Fluid Power.

[73] Pan M., Johnston N., Hillis A. (2013). Active control of pressure pulsation in a switched inertance hydraulic system. Proc. IME J. Syst. Control Eng..

[74] Akkurt N., Shedd T., Memon A.A., Ali M.R., Bouye M. (2023). Analysis of the forced convection via the turbulence transport of the hybrid mixture in three-dimensional L-shaped channel. Case Stud. Therm. Eng..

[75] Gao Q., Wang J., Zhu Y., Wang J., Wang J. (2023). Research status and prospects of control strategies for high speed on/off valves. Processes.

[76] Gao Q., Zhu Y., Wu C., Jiang Y. (2021). Identification of critical moving characteristics in high speed on/off valve based on time derivative of the coil current. Proc. IME J. Syst. Control Eng..

[77] Gao Q., Zhu Y., Wang Y. (2022). Rapid flow measurement for high speed on/off valve based on coil current derivative. J. Mech. Sci. Technol..

[78] Zhao J., Zhang C., Zhao Z., Wang Z., Yao J. (2018). Static and dynamic characteristics of high-speed on-off digital valves. China Mech. Eng..

[79] Pai-xia L.I., Xiao-jun Z., Ke-ming L.I.U. (2018). Response characteristics of high-speed on-off valve with double voltage driving. Chin. Hydraul. Pneum..

[80] Lee I.-Y. (2006). 2006 IEEE International Conference on Industrial Technology, IEEE.

[81] Zhao J., Wang M., Wang Z., Grekhov L., Qiu T., Ma X. (2017). Different boost voltage effects on the dynamic response and energy losses of high-speed solenoid valves. Appl. Therm. Eng..

[82] Zhao J., Yue P., Grekhov L., Ma X. (2018). Hold current effects on the power losses of high-speed solenoid valve for common-rail injector. Appl. Therm. Eng..

[83] Zhong Q., Zhang B., Hong H.C., Yang H.Y. (2018).

[84] Zhong Q. (2021). Performance analysis of high speed on/off valve by multi-voltages compound excitation. J. Mech. Eng..

[85] Zhong Q. (2021). Analysis of dynamic characteristics and power losses of high speed on/off valve with pre-existing control algorithm. Energies.

[86] Qiang G.A.O., Yuchuan Z.H.U., Zhang L.U.O., Xiaoming C. (2019). Analysis and optimization on compound PWM control strategy of high-speed on/off valve. 北京航空航天大学学报.

[87] Gao Q., Zhu Y., Luo Z., Bruno N. (2020). Investigation on adaptive pulse width modulation control for high speed on/off valve. J. Mech. Sci. Technol..

[88] Zhong Q., Zhang B., Yang H.-Y., Ma J.-E., Fung R.-F. (2017). Performance analysis of a high-speed on/off valve based on an intelligent pulse-width modulation control. Adv. Mech. Eng..

[89] Zhang B. (2018). Self-correcting PWM control for dynamic performance preservation in high speed on/off valve. Mechatronics.

[90] Gao Q. (2022). Investigation on the transient impact characteristics of fast switching valve during excitation stage. J. Low Freq. Noise Vib. Act. Control.

[91] Tamburrano P., Sciatti F., Distaso E., Amirante R. (2023). Comprehensive numerical analysis of a four-way two-position (4/2) high-frequency switching digital hydraulic valve driven by a ring stack actuator. Energies.

[92] Flugge-Lotz I., Taylor C. (1956). Synthesis of a nonlinear control system. IRE Trans. Automatic Control.

[93] Laamanen A., Linjama M., Vilenius M. (2003). 7th Triennial International Symposium on Fluid Control, Measurement and Visualization, Sorrento, Italy, August 25-28.2003.

[94] Linjama M. (2016). Proceedings of the Eight Workshop on Digital Fluid Power.

[95] Linjama M., Huova M., Karvonen M. (2012). The 5th Workshop on Digital Fluid Power.

[96] Rong L., Xuanyin W., Guoliang T., Fan D. (2001). Conference on Fluid Power Transmission and Control.

[97] Donkov V.H., Andersen T., Linjama M., Ebbesen M. (2020). Digital hydraulic technology for linear actuation: a state of the art review. Int. J. Fluid Power.

[98] Laamanen A., Linjama M., Vilenius M. (2005). Pressure peak phenomenon in digital hydraulic systems-a theoretical study. in Bath Workshop on Power Transmission and Motion Control (PTMC 2005).

[99] Laamanen A., Linjama M., Vilenius M. (2007).

[100] Lee J.S., Lee K.B., Lee C.G. (2001). An experimental study on the control of pressure transients using an orifice. Int. J. Pres. Ves. Pip..

[101] Tanaka H. (1988). Electro-hydraulic PCM control. J. Fluid Control.

[102] Laamanen A., Nurmia M., Linjama M., Koskinen K.T., Vilenius M. (2003). The Eight Scandinavian International Conference on Fluid Power, Proceedings of the Conference, May 7-9, 2003, Tampere, Finland, SICFP' 03.

[103] Linjama M., Vilenius M. (2004).

[104] Laamanen A., Linjama M., Vilenius M. (2006). Proceedings of the 4th FPNI-PhD Symposium.

[105] Linjama M. (2012). Proceedings of the 8th International Fluid Power Conference.

[106] Paloniitty M., Linjama M. (2018). High-linear digital hydraulic valve control by an equal coded valve system and novel switching schemes. Proc. IME J. Syst. Control Eng..

[107] Li W., Han J., Ren L. (2013). New control theory and method of the digital hydraulic cylinder. Ji Xie She Ji Yu Yan Jiu.

[108] Scheidl R., Winkler B., Kogler H., Ladner P., Haas R., Lukachev E. (2016).

[109] Hesse H., Moller H. (1972).

[110] El-Ibiary Y.M., Ukrainetz P.R., Pn N. (1978). Proceedings of 34 Th.

[111] Ichiryu K. (1984).

[112] Luo H., Yang Y., Xu L. (1987). 42th National Conference on Fluid Power.

[113] Parker G.A., Yuksel I. (1981). A Novel Electro-Hydraulic Switching Valve.

[114] Cui P., Burton R.T., Ukrainetz P.R. (1991). Development of a high speed on/off valve. SAE Trans..

[115] Yu J., Han X., Zhang Y. (1994). Application of high speed digital control solenoid valves in the electronic control of diesel engines. J. Beijing Inst. Technol. (Soc. Sci. Ed.).

[116] Kong X., Li S. (2014). Dynamic performance of high speed solenoid valve with parallel coils. Chin. J. Mech. Eng..

[117] Tu H.C., Rannow M.B., van de Ven J.D., Wang M., Li P.Y., Chase T.R. (2007). ASME International Mechanical Engineering Congress and Exposition.

[118] Gu L. (2013).

[119] Zhang J., Yang M., Xu B. (2018). Design and experimental research of a miniature digital hydraulic valve. Micromachines.

[120] Yang M., Zhang J., Xu B. (2018). Experimental study and simulation analysis on electromagnetic characteristics and dynamic response of a new miniature digital valve. Adv. Mater. Sci. Eng..

[121] Yang M., Zhang J., Xu B., Wang W. (2019). Study on electromagnetic force of the new micro digital valve. Microsyst. Technol..

[122] Sui L., Xiong X., Shi G. (2012). Piezoelectric actuator design and application on active vibration control. Phys. Procedia.

[123] Yokota S., Hiramoto K., Akutsu K. (1993). Proceedings of the JFPS International Symposium on Fluid Power.

[124] Yokota S., Akutu K. (1991). A fast-acting electro-hydraulic digital transducer: a poppet-type on-off valve using a multilayered piezoelectric device. JSME international journal. Ser. 2, Fluids engineering, heat transfer, power, combustion, thermophysical properties.

[125] Yamada H., Wennmacher G., Muto T., Suematsu Y. (2000). Development of a high-speed on/off digital valve for hydraulic control systems using a multilayered PZT actuator. Int. J. Fluid Power.

[126] Ouyang X., Yang H., Jiang H., Xu B. (2008). Simulation of the piezoelectric high-speed on/off valve. Chin. Sci. Bull..

[127] Zeng Y., Wang D., Zi B., Zeng Q. (2015). Dynamic characteristics of priority control system for high-speed on–off digital valve. Adv. Mech. Eng..

[128] Wang S., Zhang B., Zhong Q., Yang H. (2017). Study on control performance of pilot high-speed switching valve. Adv. Mech. Eng..

[129] Kogler H., Scheidl R. (2008). Two basic concepts of hydraulic switching converters. Proceedings of the First Workshop on Digital Fluid Power.

[130] Pan M., Plummer A. (2018). Digital switched hydraulics. Front. Mech. Eng..

[131] Yuan C., Pan M., Plummer A. (2020). A review of switched inertance hydraulic converter technology. J. Dyn. Syst. Meas. Control.

[132] Nordås S., Ebbesen M.K., Andersen T.O. (2018). Fluid Power Systems Technology.

[133] Zhu K., Gu L., Chen Y., Li W. (2012). High speed on/off valve control hydraulic propeller. Chin. J. Mech. Eng..

[134] Rannow M.B., Li P.Y. (2009). Dynamic Systems and Control Conference.

[135] Rickenberg F. (1930).

[136] Bower J.L. (1961). Digital fluid control system. Google Patents, Sep..

[137] Murphy R., Weil J. (1962).

[138] Virvalo T.K. (1978). Cylinder speed synchronization. Hydraul. Pneum..

[139] Linjama M., Koskinen K.T., Vilenius M. (2002). Proceedings of the JFPS International Symposium on Fluid Power.

[140] Laamanen A., Linjama M., Tammisto J., Koskinen K.T., Vilenius M. (2002). Proceedings of the JFPS International Symposium on Fluid Power.

[141] Linjama M., Vilenius M. (2005). Improved digital hydraulic tracking control of water hydraulic cylinder drive. Int. J. Fluid Power.

[142] Linjama M., Huova M., Vilenius M. (2008). Proceedings of the 6th International Fluid Power Conference Dresden.

[143] Huova M., Karvonen M., Ahola V., Linjama M., Vilenius M. (2010). 7th International Fluid Power Conference (7th IFK), 22-24.

[144] Paloniitty M., Linjama M., Huhtala K. (2014). International Fluid Power Conference.

[145] Paloniitty M., Linjama M., Huhtala K. (2015). Equal coded digital hydraulic valve system–improving tracking control with pulse frequency modulation. Procedia Eng..

[146] Linjama M., Paloniitty M., Tiainen L., Huhtala K. (2015). Mechatronic design of digital hydraulic micro valve package. Procedia Eng..

[147] Ketonen M., Linjama M. (2017). Proc. Of the Ninth Workshop on Digital Fluid Power.

[148] Linjama M., Vilenius M. (2005). Proceedings of the JFPS International Symposium on Fluid Power.

[149] Huova M., Laamanen A. (2009). Proceedings of the Second Workshop on Digital Fluid Power 12-13 November 2009.

[150] Heybroek K., Norlin E. (2015). Hydraulikdagarna, Linköping, Sweden, 16-17 March 2015 Hydraulic.

[151] De Gier G. (2004).

[152] Linjama M., Vihtanen H.-P., Sipola A., Vilenius M. (2009). The 11th Scandinavian International Conference on Fluid Power SICFP’09.

[153] Sciatti F., Tamburrano P., Distaso E., Amirante R. (2023). Journal of Physics: Conference Series.

